# Investigating the role of verbal templates in contingent capture by color

**DOI:** 10.3758/s13414-019-01701-y

**Published:** 2019-03-28

**Authors:** Diane Baier, Ulrich Ansorge

**Affiliations:** Department of Basic Psychological Research and Research Methods, Faculty of Psychology, University of Vienna, Liebiggasse 5, 1010 Vienna, Austria

**Keywords:** Color, Contingent capture, Attention capture, Search templates

## Abstract

To investigate if top-down contingent capture by color cues relies on verbal or semantic templates, we combined different stimuli representing colors physically or semantically in six contingent-capture experiments. In contingent capture, only cues that match the top-down search templates lead to validity effects (shorter search times and fewer errors for validly than for invalidly cued targets) resulting from attentional capture by the cue. We compared validity effects of color cues and color-word cues in top-down search for color targets (Experiment 1a) and color-word targets (Experiment 2). We also compared validity effects of color cues and color-associated symbolic cues during search for color targets (Experiment 1b) and of color-word cues during search for both color and color-word targets (Experiment 3). Only cues of the same stimulus category as the target (either color or color-word cues) captured attention. This makes it unlikely that color search is based on verbal or semantic search templates. Additionally, the validity effect of matching color-word cues during search for color-word targets was neither changed by cue-target graphic (font) similarity versus dissimilarity (Experiment 4) nor by articulatory suppression (Experiment 5). These results suggested either a phonological long-term memory template or an orthographically mediated effect of the color-word cues during search for color-words. Altogether, our findings are in line with a pronounced role of color-based templates during contingent capture by color and do not support semantic or verbal influences in this situation.

To accomplish successful search for visual targets, humans set up and maintain target templates in (visual) working memory (Desimone & Duncan, 1995; Hamker, 2004) or long-term memory (Carlisle, Arita, Pardo, & Woodman, 2011) and compare them with the presently seen stimuli. Selection of seen stimuli for further in-depth processing is then a function of the match between stimulus features and top-down search templates (Bundesen, 1990; Duncan & Humphreys, 1992).

However, up to now, the exact composition of search templates in visual search for colors has not been identified conclusively. Different possibilities have been discussed: Search templates might consist of feature representations in visual working memory (e.g., Baddeley, 1986; Berggren & Eimer, 2016; Daffron & Davis, 2016; Jenkins, Grubert, & Eimer, 2017; Olivers, Peters, Houtkamp, & Roelfsema, 2011; Vickery, King, & Jiang, 2005), be based on verbal representations of searched-for features in (working) memory (e.g., Lupyan & Spivey, 2010; Smyth & Scholey, 1994; Walenchok, Hout, & Goldinger, 2016; Wolfe, Horowitz, Kenner, Hyle, & Vasan, 2004), or consist of semantic representations that are shared by visual objects and words (e.g., Belke, Humphreys, Watson, Meyer, & Telling, 2008; Moores, Laiti, & Chelazzi, 2003; Schmidt & Zelinsky, 2009).

Here, we investigated the substrate of search templates in contingent capture by color. According to the contingent-capture theory, attentional capture by uninformative color cues depends on a match between cue color and top-down search templates (Folk & Remington, 1998). In each trial of a contingent-capture experiment, participants search for a predefined target at one out of several possible positions. For example, participants search for a red target that in each trial can equally likely occur at one of four positions and report the target’s shape. In this situation, target-similar, top-down matching cues presented prior to the targets capture attention: Valid cues (presented at target position) facilitate search as compared with invalid cues (presented away from the target). This validity effect is indicative of attention capture by the cue, by which valid cues capture attention to the target and invalid cues vie for attention away from the target (cf. Posner, Snyder, & Davidson, 1980). In case of top-down matching cues, the validity effect is even found if the cue is on average not predictive of the most likely target position. This is in contrast to nonmatching cues that, if uninformative, seemingly do not capture attention and do not lead to validity effects (Folk, Remington, & Johnston, 1992). For example, during search for red targets, uninformative red cues give rise to a validity effect, but green cues do not (Folk & Remington, 1998).

What is true of visual search in general is also true of contingent capture by color: Currently, it is not known what kind of template is underlying the contingent-capture effect (i.e., validity effect in matching but not nonmatching conditions) by uninformative color cues. In the current study, to investigate the structure of the top-down search templates in contingent capture by color (cue) stimuli, we combined both color stimuli, symbolic stimuli associated with a color, and color-word stimuli. Words are complex stimuli, and thus it is demanding to read them when presented extrafoveally. However, humans process words presented away from the current fixation point (e.g., Bradshaw, 1974; Kliegl, Risse, & Laubrock, 2007; Shaffer & LaBerge, 1979; Underwood, 1977), and this is true for color words, too (e.g., Gatti & Egeth, 1978; Kahneman & Chajczyk, 1983). It has also been shown that words can capture attention, even if task-irrelevant (e.g., Stein, Zwickel, Kitzmantel, Ritter, & Schneider, 2010).

In the present study, we used color words for primarily two reasons. First, color-word cues allow studying if participants use a verbal template to search for colors, to which a color-word cue denoting the searched-for color would match. Second, if participants use a semantic search template for color, again, a color-word cue denoting the searched-for color would match to the template and would capture attention. In Experiment 1b, instead of color-word cues, we used parafoveally presented symbolic shape cues associated semantically with a specific color (i.e., a heart [associated with red] or a star [associated with yellow]). Like words, these symbols would capture attention if participants use a semantic search template. In detail, color stimuli carry color information via their hues (here, as colored rings), whereas color words carry the information via their identity or meaning, and symbols via their semantic association with a specific color. If top-down templates during search for color-defined targets consist of hue representations, color-word cues and symbolic cues might be successfully ignored, even if they denote one of the searched-for colors (at least as long as they do not carry the searched-for color). However, if top-down templates during color search consist of verbal or semantic representations, color-word cues denoting the searched-for target hues or symbols being associated with the searched-for color could capture attention, just as color-ring cues of a matching color. For example, validity effects of top-down matching color-word cues could be due to the participants’ subvocal rehearsal of color names during search for target colors. In addition to such a verbally mediated contingent-capture effect, semantic representations of colors could be shared among words, symbols, and sensory features (cf. Ariga & Yokosawa, 2008; Dalrymple-Alford, 1968, 1972; Klein, 1964). According to the theory of embodied cognition (e.g., Barsalou, 1999) and to the feedback-labeling hypothesis (Lupyan, 2012), for example, conceptual semantic knowledge or even words are linked to perceptual representations, implying that color and color-word representations could reciprocally activate one another, thereby, theoretically allowing for a contingent-capture effect based on color words or symbols associated to target colors during search for color-ring targets, too. Table 1 gives an overview of the experiments and the major findings of the present study.

**Table 1:** Overview of designs and results of Experiments 1–5

			Results	
Exp.	Cues	Targets	RTs	ACCs
1a	Colored rings & color words	Colored rings	Contingent-capture effects only by color-ring cues, not by color-word cues	Contingent-capture effects only by color-ring cues, not by color-word cues
1b	Colored rings & symbols	Colored rings	Contingent-capture effects by color-ring cues and inversed validity effect by matching symbols	Contingent-capture effects only by color-ring cues, not by symbolic cues
2	Colored rings & color words	Color words	Validity effects only by matching but not by nonmatching color-word cues, and not by color-ring cues	Validity effects by matching but not by nonmatching color-word cues, and small validity effect by matching color-ring cues
3	Color words	Colored rings & color words	Validity effects by matching but not by nonmatching color-word cues (preceding color-word targets); validity effect by any color-word cues preceding color-ring targets	No significant effects
4	Color words with target-similar or target-dissimilar font	Color words	Validity effects by matching but not by nonmatching color-word cues independent of cue–target font similarity	Validity effects by matching but not by nonmatching color-word cues independent of cue–target font similarity
5	Color words & additional articulatory suppression or foot tapping	Color words	Validity effects by matching but not by nonmatching color-word cues independent of additional task	Validity effects by matching but not by nonmatching color-word cues independent of additional task

*Note*. RT = reaction time, ACC = accuracy

## Experiment 1a

In Experiment 1a, to differentiate between verbal/semantic and hue-based search templates, we used both color cues and color-word cues during search for a color-ring target. Cues were not predictive of the most likely target position, and, therefore, only cues that matched the search template for the color-ring target should capture attention (Folk & Remington, 1998; Goller, Ditye, & Ansorge, 2016). If verbal or semantic templates play a role in visual search for color-ring targets, color-ring cues as well as color-word cues should lead to contingent-capture effects: validity effects where the color-ring cue or the color-word cue matched the target color and no or smaller validity effects where the color-ring or the color-word cue did not match the target color. In contrast, if search templates are based on sensory hue representations, only color-ring cues but not color-word cues should lead to contingent-capture effects because color-word cues were presented in a nonmatching color. (The latter was necessary to prevent contingent capture based on the hues of the color-word cues alone.)

### Method

#### Participants

Twenty-one psychology students from the University of Vienna participated in Experiment 1a (*M*_age_ = 21.00 years, *SD*_age_ = 1.69 years). The students received course credit points for their participation. Their vision and color vision were normal or corrected to normal. The treatment of all participants was in line with established ethical standards: Prior to testing, participants signed an informed consent form. They were instructed thoroughly and knew that they could abort the experiment at all times without any negative consequences. The participants were neither deceived nor harmed in any way, and their well-being was monitored and ensured throughout the testing. Data collection was fully anonymous. Each student participated in only one of the following six experiments. The minimum sample size of 18 participants to detect contingent-capture effects with sufficient statistical power was estimated in a power analysis (α = .05, β = .2, *f*^2^ = 0.5). The large effect size (Cohen, 1988) was chosen in line with results from prior contingent-capture studies (cf. Büsel, Voracek, & Ansorge, 2018a; Schoeberl, Ditye, & Ansorge, 2017).

#### Apparatus and stimuli

Stimuli were presented on a 19-in. LCD monitor (Acer B 193), with an aspect ratio of 3:4, a resolution of 1,280 × 1,024 pixels, and a vertical refresh rate of 75 Hz. The graphic card was an Nvidia GeForce (GT 220, color 32 bit/96 DPI).

Participants sat in front of the screen, with a distance of 57 cm. Their head rested on a chin rest. The room was indirectly and dimly lit by small USB-powered lamps behind the screens. Up to four participants were tested simultaneously. The responses were given with the two index fingers pressing keys *F* and *J* of a standard keyboard, respectively, depending on the orientation of the target letter *T* (see below). The experiment was programmed and conducted using E-Prime 2.0 (Psychology Software Tools, Pittsburgh, PA).

The size of the stimuli was 1.5° × 1.5° of visual angle, with a distance of 3.0° of visual angle between stimuli. In each trial, four stimuli were presented simultaneously in the upper left, upper right, lower left, and lower right corners of a virtual square around screen center. In the target displays, one small tilted *T* (0.5° × 0.5° visual angle) was shown in white per each of the four positions (centered on these positions). Two of these letters *T* were tilted to the left, and two were tilted to the right. Each such letter was surrounded by a colored ring.

**Fig. 1:**
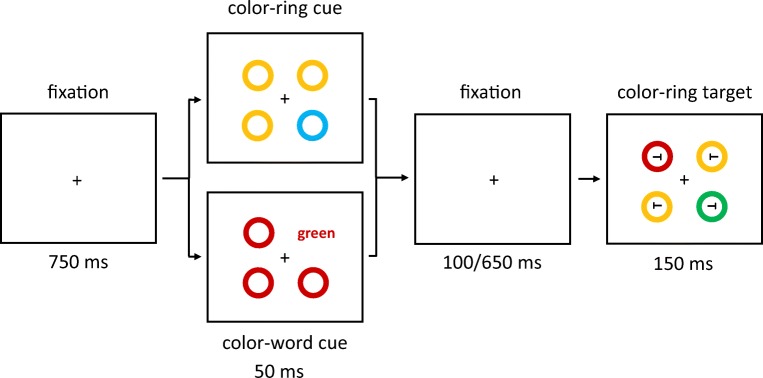
Exemplary trial(s) of Experiment 1a. The target is the green ring in the rightmost box (the target remained either green or blue, varied between participants, throughout the whole experiment). Two different cue versions are displayed in the boxes second from left: valid nonmatching color-ring cue (top); invalid matching color-word cue (bottom). In the experiment, German words were used. The arrows depict the flow of time. Stimuli are not drawn to scale. (Color figure online)

Prior to the target displays, a cueing display was shown. In half of the trials, the cueing display consisted of four colored rings (all without letters *T*). The cue ring was presented as a color singleton between three color-homogenous rings. In the other half of trials, in the cueing displays, one of the four rings was replaced by a color-word stimulus that consisted of roughly the same number of pixels as a ring. To maintain as much similarity as possible between color-ring cue and color-word cue conditions, the nonsingletons in the cueing displays were colored rings in both of these conditions, and only the cues were different. See Fig. 1.

The colors of the stimuli were green (CIE L*a*b*, 73.0, −82.7, 52.9), blue (73.1, 15.4, −119.7), red (72.8, 97.3, 93.8), and yellow (73.0, −7.1, 60.2). All colors were equiluminant and presented against a gray background (85.0, −4.8, −21.5).

All statistical analyses were conducted using R (R Core Team, 2017) and the following packages: *apa* (Gromer, 2017), *ez* (Lawrence, 2016), *ggplot2* (Wickham, 2009), and *pwr* (Champely, 2017).

#### Task and design

Experiment 1a consisted of four within-subjects variables: cue type (color-ring cue, color-word cue), cue match (matching, nonmatching), validity (valid, invalid), and stimulus-onset asynchrony (SOA) between cue and target (150 ms, 700 ms). The cue was uninformative (25% valid and 75% invalid), and cue and target positions were randomized and uncorrelated across trials.

The target was a predefined color ring in green or blue (balanced across participants). It was presented either among one red and two yellow distractors or among two red and one yellow distractor at the remaining target-display positions. The target was thus never a singleton. This was done to prevent top-down singleton search (Bacon & Egeth, 1994). In contrast to the targets, all cues were singletons presented among the cueing displays’ nonsingleton rings. The color-ring cues stood out by their unique color among the color-homogeneous nonsingleton rings. The German color-word cues were words that stood out by their shape and their meaning among the shape-homogeneous nonsingleton rings. In both color-ring cue and color-word cue displays, the nonsingleton rings were either all red or all yellow. The matching color-ring cue had the same color as the target. For the participants for which the target was blue, the matching color-ring cue was blue, and for the participants for which the target was green, the matching color-ring cue was green. In contrast, the nonmatching color-ring cue was blue if the target was green, and it was green if the target was blue. If a color-word cue was presented, it was of the same irrelevant font color (red or yellow) as the remaining nonsingleton rings. A potentially matching color-word cue denoted the same color as the searched-for target color (e.g., the German word *grün*, meaning *green*, if the target was green). A potentially nonmatching color-word cue denoted the same color as was used for the nonmatching color-ring cue (e.g., the German word *blau*, meaning *blue*, if the target was green). This was done to prevent that the color-word cue could have captured attention by means of its top-down matching color or by its status as a color singleton alone.

Participants had to search for the target and respond to the orientation of the tilted *T* inside of the target ring. Orientation-to-response key mappings were counterbalanced across participants. Figure 1 shows exemplary trials of Experiment 1a.

#### Procedure

Each trial started with a fixation cross, centrally located on the screen. After 750 ms, the cueing display was presented. It consisted of one singleton-color-ring cue or one color-word cue and three nonsingleton rings presented for 50 ms. Stimuli in the cueing displays were pseudorandomly assigned to the four positions.

After an SOA of 150 or 700 ms, the target display was presented. It consisted of a target and three distractors that were displayed at the same four positions for 150 ms (see Fig. 1). Like the cues and nonsingletons, target and distractors were pseudorandomly assigned to the four positions. The two different SOAs were chosen after Wolfe, Butcher, Lee, and Hyle (2003). The shorter SOA might facilitate the effects of color-ring cues (cf. Gibson & Amelio, 2000), whereas the longer SOA might facilitate the effects of color-word cues, as reading words might take longer than perceiving colors.

Trials with matching and nonmatching color-ring cues and trials with potentially matching and nonmatching color-word cues were equally likely and presented in a pseudorandom sequence. Prior to data collection, participants received written and verbal instructions explaining the task and practiced until they made fewer than 20% errors. During the experiment, no feedback was provided about the correctness of the responses. Together with 768 experimental trials, the whole experiment took approximately 60 min.

### Results

Initially, to check for advantages in perception and processing of word cues presented on the right side that could have been due to more parafoveal preview benefits for word cues on the right versus left, we conducted an analysis of variance (ANOVA), including cue-side as an independent variable. This variable did not show more cueing by word cues on the right. For all further analyses, we therefore collapsed data across cue sides.

#### Reaction-time analysis

Trials with false responses were removed, as well as responses faster or slower than two standard deviations from the median per person and condition (in total, 9.79%). Although a two-standard-deviation cutoff is quite common (e.g., Ansorge, Kiss, Worschech, & Eimer, 2011), we additionally analyzed all data with a more liberal cutoff (± three *SD*s per person per condition). The pattern of the results remains the same, except for some minor differences (for a detailed listing of the differences, refer to Appendix A). A 2 × 2 × 2 × 2 repeated-measures ANOVA of the correct mean reaction times (RTs), with the variables cue type (color-ring cue, color-word cue), validity (valid, invalid), cue match (matching, nonmatching), and SOA (150 ms, 700 ms), was conducted. Here and in the following experiments, only the significant highest order interaction is reported. For all significant main effects and lower order interactions that were qualified by the highest order interaction, the interested reader should refer to Appendix B. A complete listing of mean correct RTs and accuracy rates for all conditions and experiments can be found in Appendix C.

Contingent-capture effects show as an interaction of validity and cue match in the ANOVA, as the validity effects are selective (only top-down matching cues lead to validity effects). To pinpoint the interaction of validity and cue match, matching valid versus invalid conditions (for which significant validity effects are expected), as well as nonmatching valid versus invalid conditions (for which no effects or same-location costs are expected) are compared in post hoc *t* tests, here, and in the following experiments. These *t* tests were conducted for each cue type and each SOA in turn if a significant three-way or four-way interaction indicated that this was necessary. For additional comparisons of matching valid versus nonmatching valid cues, as well as between matching invalid versus nonmatching invalid cues, please refer to Appendix D.

**Fig. 2:**
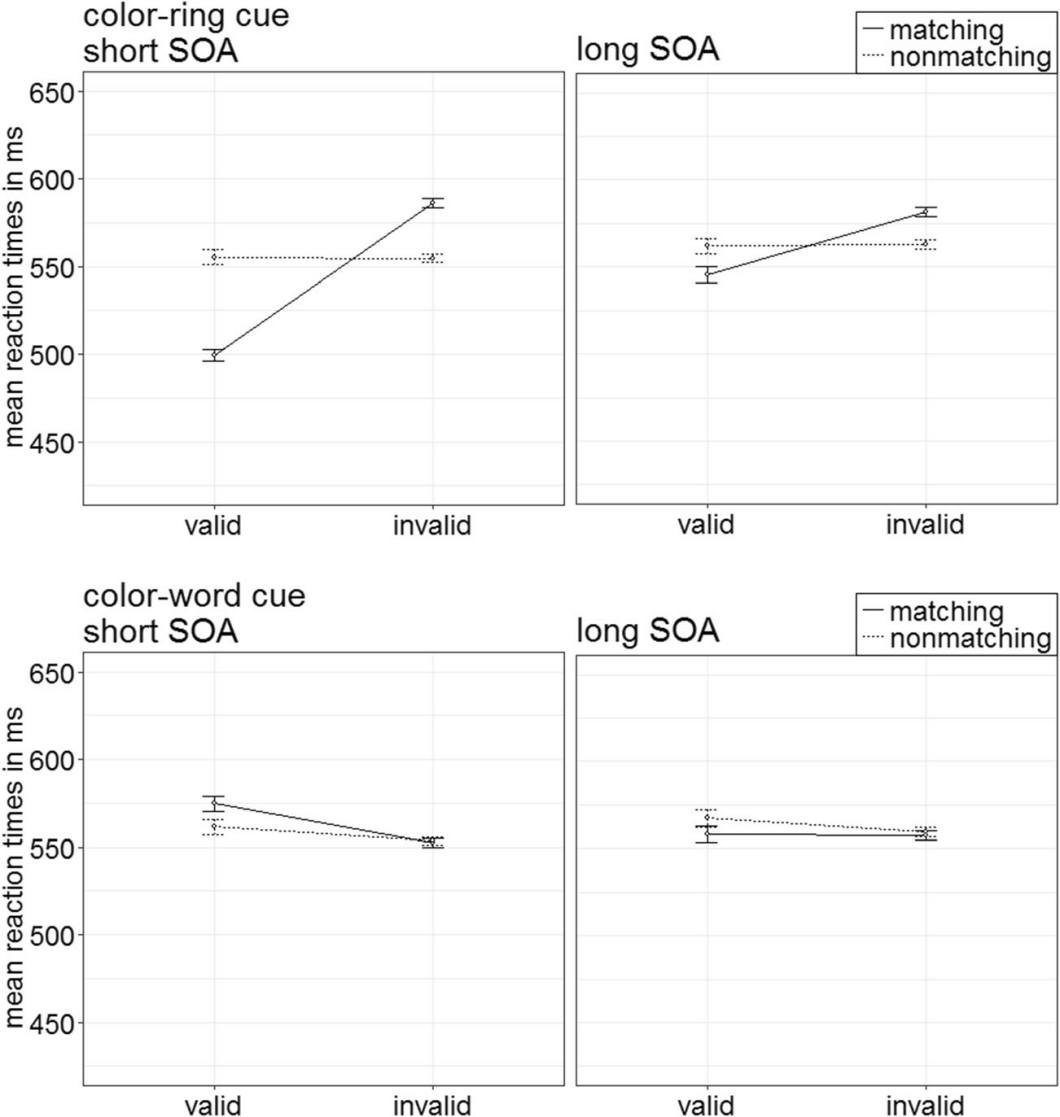
Experiment 1a: Mean correct reaction times (in ms) for color-ring cues (upper panels) and color-word cues (lower panels) depending on stimulus-onset asynchrony (SOA), cue match, and validity. Error bars represent average standard errors

The ANOVA yielded a significant four-way interaction of Cue Type × Validity × Cue Match × SOA, *F*(1, 20) = 17.75, *p* < .001, ${\upeta}_{\mathrm{p}}^2$ = .47 (see Fig. 2; Table 5 in Appendix C; other significant main effects and interactions are listed in Table 2 in Appendix B). For the follow-up *t* tests, the critical alpha of *p* = .05 was Bonferroni corrected—that is, it was divided by the number of tests (i.e., by 16), and thus alpha was .003.

##### Color-ring cues

The *t* tests revealed contingent-capture effects for color-ring cues in the short SOA: faster RTs in valid (499 ms) than in invalid (586 ms) trials after a matching cue, *t*(20) = −13.84, *p* < .001, *d* = −2.95, and no validity effects with nonmatching cues, *t*(20) = 0.02, *p* = .982, *d* < 0.01. Also in the long SOA, RTs with matching color-ring cues were significantly faster in valid (546 ms) than in invalid (582 ms) conditions, *t*(20) = −4.23, *p* < .001, *d* = −0.90. Nonmatching cues did not produce any significant effects, *t*(20) = −0.49, *p* = .627, *d* = −0.11.

##### Color-word cues

Color-word cues did not show any significant effects in the post hoc tests, all nonsignificant |*t*s|(20) < 3.29, all *p*s > .004, all |*d*s| < 0.70.

#### Accuracy analysis

**Fig. 3:**
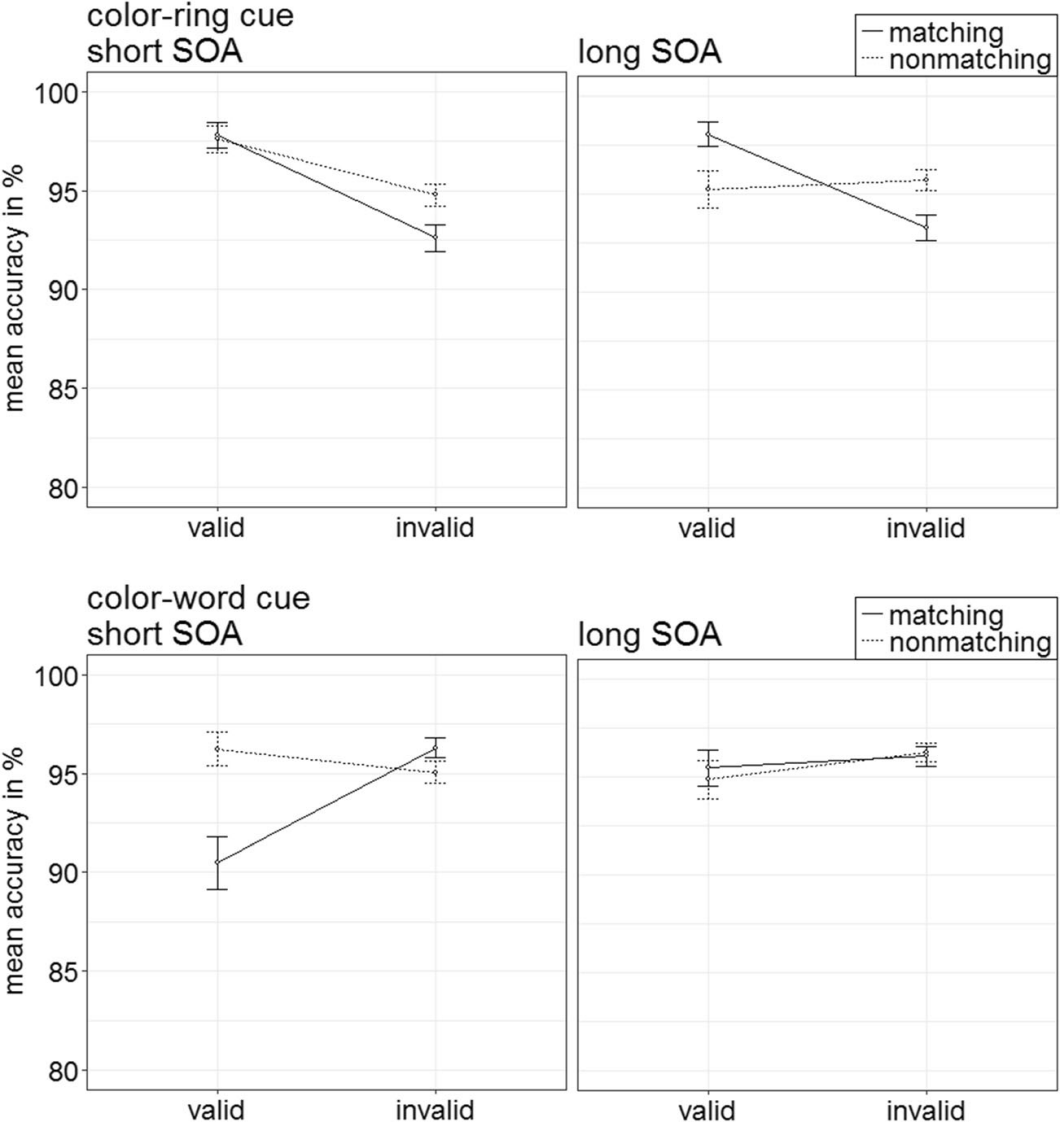
Experiment 1a: Mean accuracy rates in percentages for color-ring cues (upper panels) and color-word cues (lower panels) depending on stimulus-onset asynchrony (SOA), cue match, and validity. Error bars represent average standard errors

Per each condition, the individual rates of accurate responses were arcsine-transformed before conducting a repeated-measures ANOVA analogous to the one above. It showed a significant three-way interaction of Cue Type × Validity × Cue Match, *F*(1, 20) = 23.18, *p* < .001, ${\upeta}_{\mathrm{p}}^2$ = .54, that mirrored important results of the RT analysis (see Fig. 3). (For other significant main effects and interactions, refer to Table 3 in Appendix B. Table 5 in Appendix C shows the mean accuracy rates of all conditions.) Post hoc Bonferroni-corrected *t* tests (alpha corrected for eight comparisons, equal to .006) examined contingent-capture and validity effects for the different cue types. Here and in the following experiments, we present comparisons between valid and invalid matching, as well as nonmatching cues. For comparisons between matching and nonmatching valid, as well as matching and nonmatching invalid cues, please refer to Appendix E.

##### Color-ring cues

*T* tests yielded validity effects of matching color-ring cues, with better performance in valid (97.9%) than in invalid (92.9%) trials, *t*(20) = 6.65, *p* < .001, *d* = 1.42. Validity effects were selectively present for matching cues, and nonmatching cues did not lead to a significant validity effect, *t*(20) = 1.22, *p* = .236, *d* = 0.26.

##### Color-word cues

In the post hoc tests, word cues did not lead to any significant validity effects, all nonsignificant |*t*s|(20) < 2.87, all *p*s > .009, all |*d*s| < 0.61.

### Discussion

In Experiment 1a, we wanted to distinguish between contingent-capture effects based on templates for sensory hues versus verbal/semantic search templates by using both color-ring and color-word cues during search for a color-ring target. As expected, the color-ring cues led to significant contingent-capture effects in the short SOA condition: Only cues in a top-down matching color elicited validity effects—namely, faster reactions and fewer errors in valid than in invalid conditions. In the long SOA condition, the contingent-capture effects of color-ring cues were still present but less pronounced than in the short SOA condition (see also Experiment 5 of Ansorge, Priess, & Kerzel, 2013; Gibson & Amelio, 2000). In contrast, color-word cues led to neither contingent-capture nor to validity effects. Given that the color-word cues apparently did not capture any attention at all, the results of Experiment 1a tentatively speak for search templates consisting of sensory hue feature representations and provide no evidence for verbally or semantically mediated contingent-capture effects during search for color-ring targets. These results might be surprising, as they seem inconsistent with some other findings: For example, seeing a word alone automatically included color representations in search templates in the studies of Nako, Smith, and Eimer (2016) or R. Wu et al. (2013).

However, in Experiment 1a, the lack of capture effects by color-word cues could have been due to a failure to recognize the words. Words are complex stimuli but were presented parafoveally, and thus it might have been difficult for the participants to read them (but see, e.g., Gatti & Egeth, 1978). In Experiment 1a, the lack of capture effects by color-word cues could have also been due to other forms of generally higher processing difficulty for word cues compared to color-ring cues. The color-word cues were written in an irrelevant and incongruent font color (red or yellow). Reading color words with a font color not matching the meaning of the color word might be difficult (Durgin, 2000; but see Stroop, 1935). Additionally, participants might have actively suppressed the processing of words due to the words’ irrelevant (nonmatching) font colors. As explained in the Method section, words were presented in the same color as the distractors in the target displays. This might have increased participants’ active inhibition of stimuli that carried the corresponding colors—here, of the color-word cues. Moreover, when it comes to influences on visual attention, word cues are generally less effective than picture cues (see, e.g., Nako et al., 2016; Wolfe et al., 2004; R. Wu et al., 2013).

To control for the influence of processing duration, in Experiment 1b, we used symbolic cues associated with a specific color instead of color-word cues. If the higher processing difficulty of color words in Experiment 1a prevented that we could demonstrate a semantically based capture effect, then the use of symbolic cues in Experiment 1b might allow semantic effects to show, as these cues consisted of a single symbol rather than of a string of several letters.

## Experiment 1b

To examine the influence of semantics in top-down search templates for color, we compared color-ring cues and parafoveally presented symbolic cues in Experiment 1b. The symbolic cues were semantically associated with a specific color: a heart symbol for the color red and a star symbol for the color yellow. If the search templates were at least partly based on semantics, both the color-ring cues as well as the symbolic cues should capture attention—if they matched the searched-for color—and lead to contingent-capture effects.

### Method

#### Participants

In Experiment 1b, 23 psychology students of the University of Vienna participated (*M*_age_ = 20.04 years, *SD*_age_ = 1.92 years). Three had to be excluded due to an error rate exceeding 20%.

#### Procedure and design

We adapted the design of Experiment 1a in two ways: First, we replaced the color-word cues by symbolic cues associated with a specific color. Second, instead of green as a target color and blue as a nonmatching color (and vice versa for half of the participants), we used red as a target color and yellow as nonmatching color (and vice versa for half of the participants). We changed the color setup, as we needed symbols clearly associated with a color, and according to an online survey via Clickworker (https://www.clickworker.de), the closest associations were between red and heart (100% accordance) and yellow and star (94% accordance). To check if the symbols were indeed semantically associated with a color, we included a short congruency test after the main experiment, where participants had to discriminate the symbols (star or heart) by key press as fast as possible. These symbols had either a color congruent to their semantic association (red heart, yellow star) or incongruent (yellow heart, red star). We would expect faster responses in congruent than in incongruent trials (Ménard-Buteau & Cavanagh, 1984; Naor-Raz, Tarr, & Kersten, 2003). Every trial of the color-association test started with a fixation cross presented for 1,500 ms, followed by the colored symbol (heart or star, in red or yellow) for 50 ms, and a structural mask for 100 ms. This additional task had 48 trials (including eight practice trials).

**Fig. 4:**
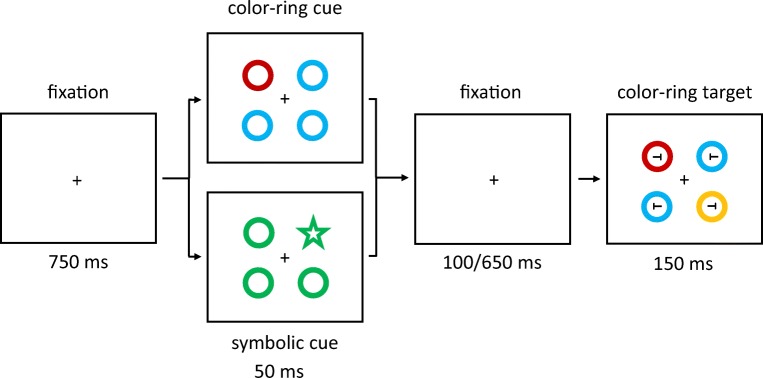
Exemplary trial(s) of Experiment 1b. The target is the red ring in the rightmost box (the target remained either red or yellow, varied between participants, throughout the whole experiment). Two different cue versions are displayed in the boxes second from left: valid matching color-ring cue (top); invalid nonmatching symbolic cue (bottom). The arrows depict the flow of time. Stimuli are not drawn to scale. (Color figure online)

The main experiment consisted of four within-subjects variables: cue type (color-ring cue, symbolic cue), cue match (matching, nonmatching), validity (valid, invalid), and SOA between cue and target (150 ms, 700 ms). Note that only one level of the factor cue type was changed compared with Experiment 1a (symbolic cues instead of color-word cues). The cueing displays with color-ring cues showed four colored rings: one color-ring cue in the target color or nonmatching color (red or yellow) and three irrelevant distractors (color rings, all either green or blue). In the condition with symbolic cues, the only difference was that instead of the color-ring cue in red or yellow, a heart or star was presented in the same color as the three irrelevant distractor rings (blue or green). This symbol could either semantically match the target color (heart for a red target, star for a yellow target) or not (star for a red target, heart for a yellow target). See Fig. 4 for an exemplary trial of Experiment 1b. Target colors were counterbalanced across participants (each participant searched for either red or yellow targets throughout the whole experiment). We expected contingent-capture effects for both color-ring cues and symbolic cues if search templates are at least partly based on semantic representations.

### Results

#### Reaction-time analysis

**Fig. 5:**
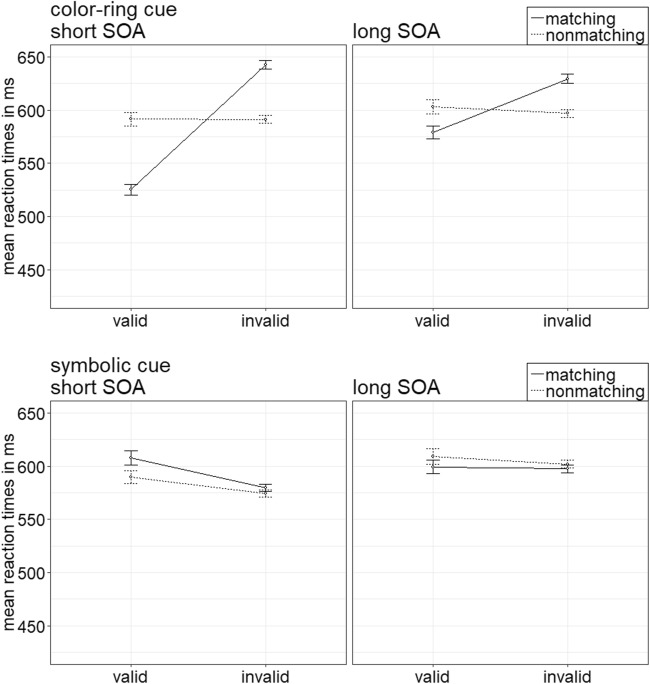
Experiment 1b: Mean correct reaction times (in ms) for color-ring cues (upper panels) and symbolic cues (lower panels) depending on stimulus-onset asynchrony (SOA), cue match, and validity. Error bars represent average standard errors

Trials with false responses were removed, as well as responses faster or slower than two standard deviations from the median per person and condition (in total 13.85%). A 2 × 2 × 2 × 2 repeated-measures ANOVA of the correct mean reaction times (RTs), with the variables cue type (color-ring cue, symbolic cue), validity (valid, invalid), cue match (matching, nonmatching), and SOA (150 ms, 700 ms), was conducted. It showed a significant four-way interaction of Cue Type × Validity × Cue Match × SOA, *F*(1, 19) = 10.15, *p* = .005, ${\upeta}_{\mathrm{p}}^2$ = .35 (see Fig. 5; Table 6 in Appendix C). For a complete listing of all significant interactions and main effects, see Table 2 in Appendix B.

We calculated post hoc *t* tests examining contingent-capture and validity effects for the different cue types and SOAs (Bonferroni correction of the critical alpha, *p* = .003).

##### Color-ring cues

We found contingent-capture effects for color-ring cues in the short SOA: faster RTs in valid (525 ms) than in invalid (643 ms) trials after a matching cue, *t*(19) = −8.04, *p* < .001, *d* = −1.75, and no validity effects with nonmatching cues, *t*(19) = −0.06, *p* = .951, *d* < −0.01. Accordingly, in the long SOA, matching cues led to faster RTs if valid (579 ms) than if invalid (629 ms), *t*(19) = −4.81, *p* < .001, *d* = −1.05, and no difference was found for nonmatching cues, *t*(19) = 0.99, *p* = .334, *d* < 0.22.

##### Symbolic cues

In the short SOA, invalid matching cues led to significantly faster reactions (580 ms) than valid matching cues (606 ms), *t*(19) = 3.72, *p* = .001, *d* = 0.81. Nonmatching cues did not produce a significant effect, *t*(19) = 2.81, *p* = .011, *d* = 0.61. In the long SOA, symbolic cues did not show any significant validity effects in the post hoc tests, all |*t*s|(19) < 0.77, all *p*s > .448, all |*d*s| < 0.17.

#### Accuracy analysis

**Fig. 6:**
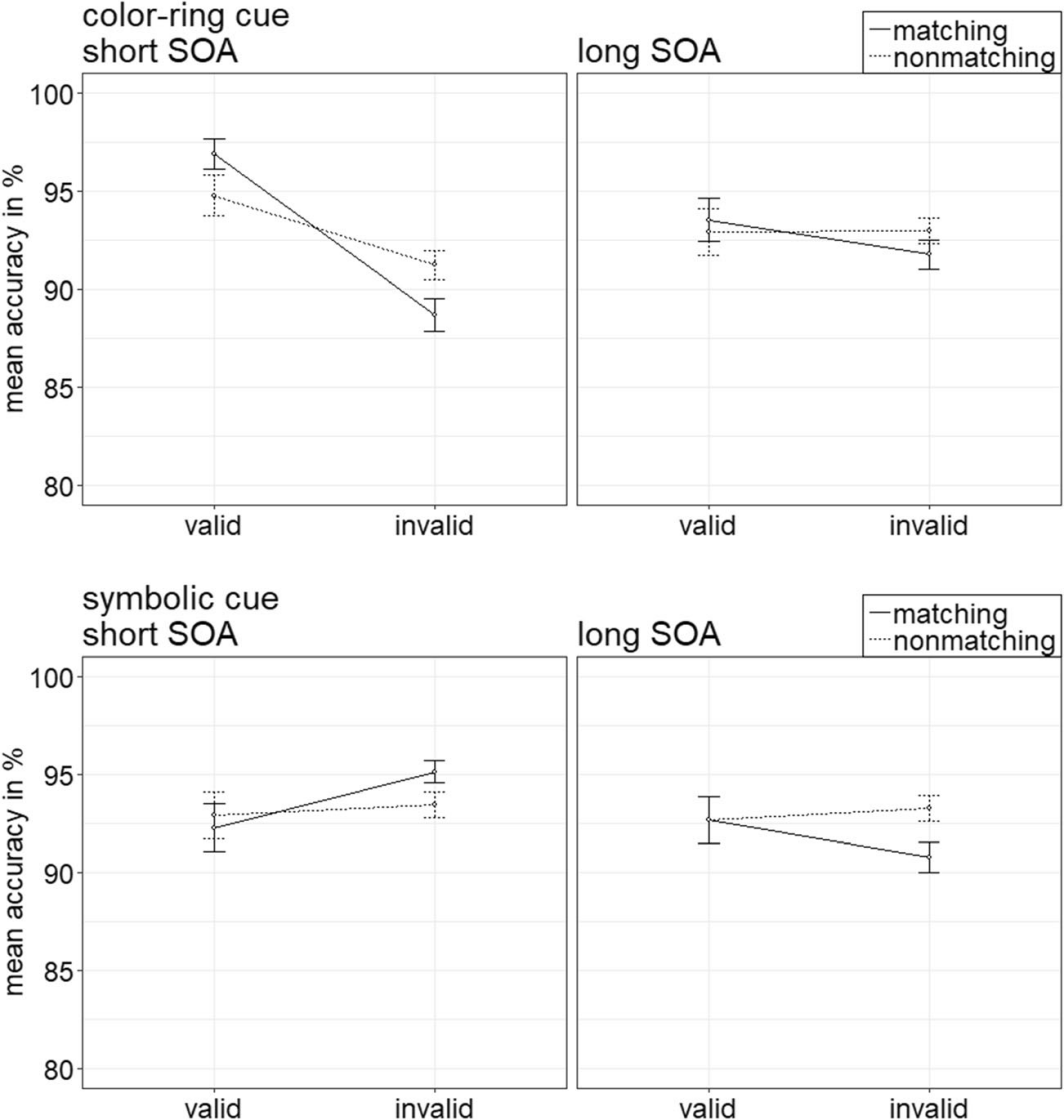
Experiment 1b: Mean accuracy rates in percentages for color-ring cues (upper panels) and symbolic cues (lower panels) depending on stimulus-onset asynchrony (SOA), cue match, and validity. Error bars represent average standard errors

Before conducting a repeated-measures ANOVA analogous to the one above, the individual accuracy rates were arcsine transformed per condition. The ANOVA yielded a significant four-way interaction of Cue Type × Validity × Cue Match × SOA, *F*(1, 19) = 5.41, *p* = .031, ${\upeta}_{\mathrm{p}}^2$ = .22 (see Fig. 6; Table 6 in Appendix C). For a complete listing of all significant interactions and main effects, see Table 3 in Appendix B. Post hoc *t* tests (with alpha Bonferroni corrected, equal to .003) examined contingent-capture and validity effects for the different cue types.

##### Color-ring cues

*T* tests revealed a contingent capture effect of color-ring cues in the short SOA condition: better performance in valid (96.9%) than in invalid (88.7%) trials, *t*(19) = 7.28, *p* < .001, *d* = 1.59, selectively for matching cues, and no significant difference between nonmatching valid and invalid cues, *t*(19) = 3.06, *p* = .006, *d* = 0.67. In the long SOA, no validity effects were found, all |*t*s|(19) < 1.79, all *p*s > .102, all |*d*s| < 0.37.

##### Symbolic cues

In the post hoc tests, symbolic cues did not lead to any significant validity effects, all nonsignificant |*t*s|(19) < 2.56, all *p*s > .019, all |*d*s| < 0.56.

#### Analysis of association between symbol and color

To check for congruency effects between symbol and associated color, we conducted a *t* test comparing RTs and accuracies to symbols in congruent versus incongruent colors. Only correct responses as well as responses within two standard deviations from the mean per person per condition were included in the analysis of the RTs (removal in total: 14.64%). Reactions were significantly faster in congruent (535 ms) than in incongruent (553 ms) conditions, *t*(19) = −2.21, *p* = .040, *d* = −0.48. Regarding accuracies, participants made fewer errors for congruently (6.75%) than for incongruently (7.75%) colored symbols. However, this difference is not significant, *t*(19) = 0.25, *p* = .606, *d* = 0.11.

### Discussion

In Experiment 1b, we presented color-ring cues and symbolic cues to find out if search templates for a color rely only on feature representations (hue) or are influenced by semantics. We found significant contingent-capture effects for color-ring cues: validity effects for top-down matching cues, no effects for nonmatching cues. The symbolic cues, however, did not produce classic contingent-capture effects, although their semantic association with a specific color was verified by congruency effects in an additional block at the end of the experiment. This would again argue for solely feature-based templates. However, in the RTs of the short SOA condition, an inversed validity effect of matching symbolic cues was significant: faster reactions after an invalid than a valid cue. This might be due to our design, where all symbols were presented in an irrelevant color (either green or blue), and, therefore, a perceptual inhibition of incongruently colored objects (e.g., a green star; Ménard-Buteau & Cavanagh, 1984) occurred. As this inhibition only arose for semantically matching symbols, semantics seem to be involved. However, the inverse validity effect of the matching symbolic cues might have had more to do with the fact that participants generally inhibited the elicitation of a color representation by the symbols that would have not been the target color in half of the trials than with the search templates for the color targets themselves. Such an active suppression of the symbols would then have had the unwanted consequence that processing of a target of the same color and at the same position as the symbolic cue would have suffered. Clearly, such an inhibition suggested by the symbolic cues would not be evidence that search for the color targets is based on a semantic template.

As not only the color-word cues in Experiment 1a but also the symbolic cues in Experiment 1b did not lead to classic contingent-capture effects, it is unlikely that the higher processing difficulty of words in Experiment 1a caused the lack of effects. This is not to deny the possibility that top-down search could be based on semantics in other instances of visual search. Instead, we think that some characteristic of the contingent-capture protocol, such as the use of uninformative cues to measure the capture of attention, might have been responsible for why participants did not use a semantic search template in Experiments 1a and 1b. To note, during search for hue-defined targets, a hue-based template would have allowed finding the targets and successfully ignoring all color-word cues—a strategy that would have made sense given that all cues were uninformative (cf. Mast & Frings, 2014). In fact, some researchers argued that cueing effects by uninformative top-down matching cues are just residual capture withstanding the participants’ general suppression of any uninformative cue, be it a nonmatching or a matching cue (Fuchs, Theeuwes, & Ansorge, 2013). In other words, whether participants use or do not use a semantic template might also depend on the details of the experimental protocol.

To control for these influences, as well as for those of word-color inconsistency, the potentially longer processing time for words than for colors, the potential difficulty of reading parafoveally presented words, and to test if verbal or semantic representations could at least in principle account for contingent-capture effects by color (cue) stimuli, in Experiment 2, we used color-word targets instead of color-ring targets. With color-word targets, color-word cues were task relevant and should be included in the search templates. As long as parafoveally presented words in the current study can be read at all, this manipulation should ensure that a validity effect can be measured at least with the matching color-word cues, even if other factors, such as word-font color inconsistency and the irrelevance of the word-cues’ font color would invite less processing of the color-word cues. During search for color-word targets, we can also test if color-ring cues capture attention if their color hue corresponds to the meaning of the searched-for color-word target.

## Experiment 2

To examine top-down search templates, in Experiment 2, we again compared color-ring and color-word cues. To avoid that participants disregarded color-word cues as irrelevant in all conditions, we replaced the color-ring targets of Experiment 1a with color-word targets. If search templates can, at least in principle, be set up for words, and parafoveally presented words can be read to a sufficient extent, then color-word cues should elicit validity effects, at least where the color-word cues corresponded to the color-word targets, and maybe not, or less so, where the color-word cues do not correspond to the searched-for color-word targets. Furthermore, if search for colors can, in principle, be carried out by verbal or semantic search templates, then color-ring cues with a color matching versus not matching the meaning of the color-word targets might again show contingent-capture effects. However, if successful selection of cues by their colors critically depends on visual search templates for sensory hue features, then color-ring cues with a hue matching versus not matching the meaning of the color-word targets might not show a contingent-capture effect, and only matching color-word cues might lead to validity effects.

### Method

#### Participants

Twenty psychology students (*M*_age_ = 23 years, *SD*_age_ = 3.43 years) of the University of Vienna participated in Experiment 2. Two students had to be excluded due to an error rate exceeding 20%.

#### Procedure and design

**Fig. 7:**
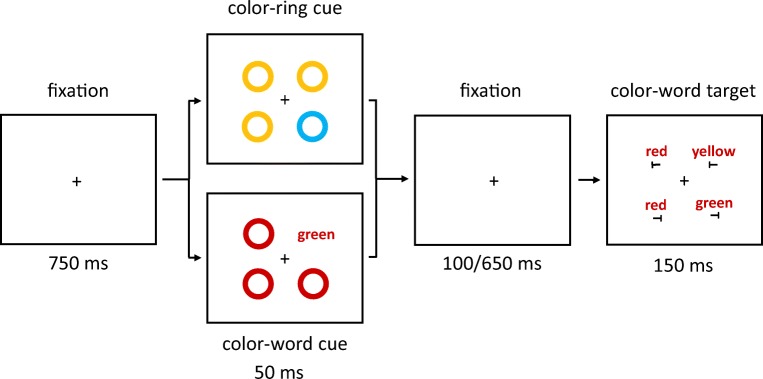
Exemplary trial(s) of Experiment 2. The target is the color word *green* in the rightmost box (the target word remained either *green* or *blue*, varied between participants, throughout the whole experiment). Two different cue versions are displayed in the boxes second from left: valid nonmatching color-ring cue (top); invalid matching color-word cue (bottom). The arrows depict the flow of time. Stimuli are not drawn to scale. (Color figure online)

The major modifications concerned the target displays: Participants searched for a predefined color-word target instead of a colored ring. Half of the participants searched for the target word *blue*, and the other half searched for the target word *green*. In addition to the color-word target, three color-word distractors (denoting the irrelevant colors red and yellow) were presented simultaneously. In the target displays, the font color of all color words, target and distractors, was the same and irrelevant. In half of the trials, all words in the target displays were red, and in the other half of the trials all words in the target displays were yellow. In addition, to prevent shape-singleton search and ensure that participants had to read the color-word targets, either two of the target displays’ distractor words read *yellow* and one read *red*, or two of the distractor words read *red* and one read *yellow*. Finally, in the target displays, all color words were presented slightly above the center of each potential target position, and all tilted *T*s were shown slightly below the center of each corner of the virtual square surrounding the screen center, as to allow for a good view of color words and tilted *T*s presented at the same time. Everything else, including the different exact types of cueing displays, remained unchanged from Experiment 1a (see Fig. 7).

### Results

#### Reaction-time analysis

**Fig. 8:**
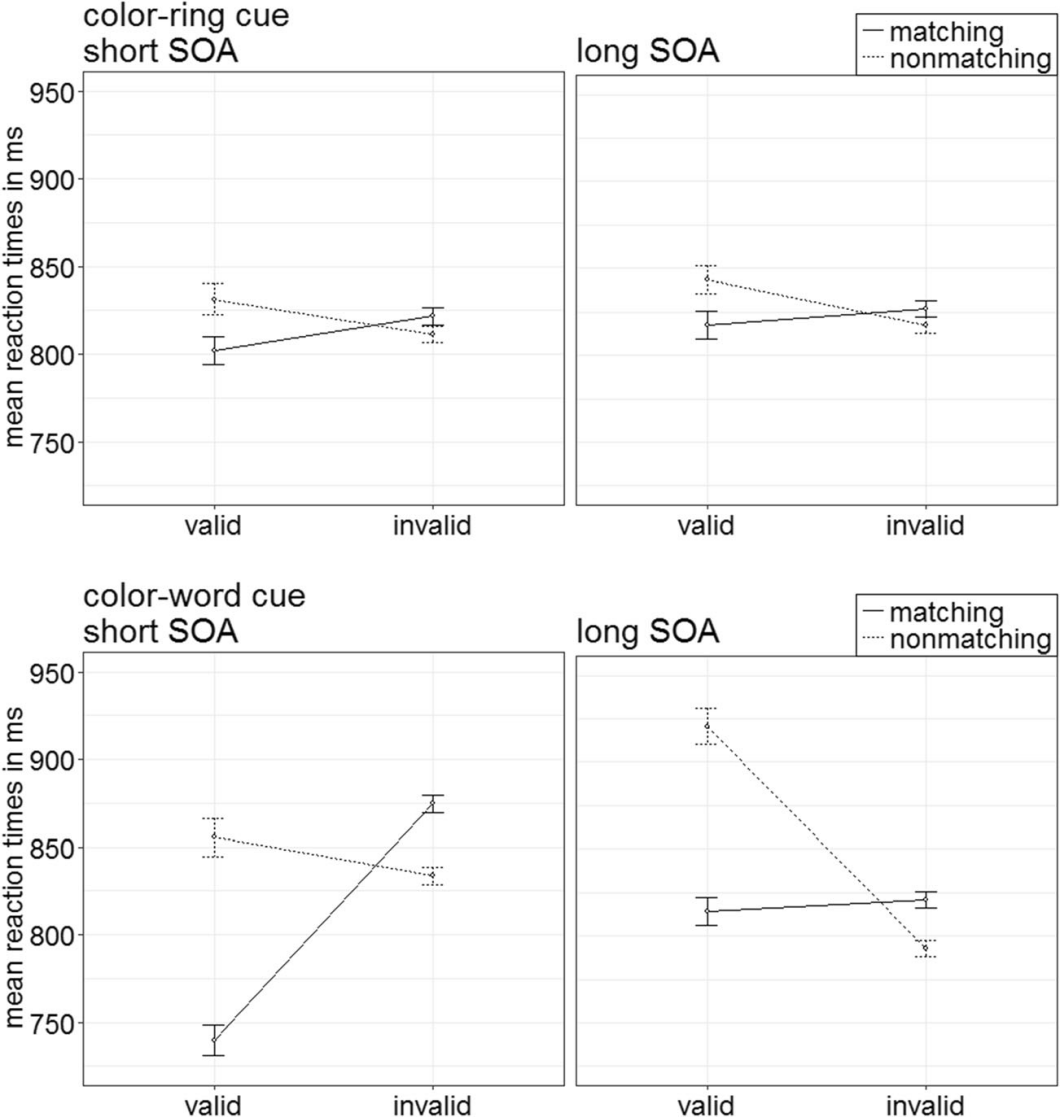
Experiment 2: Mean correct reaction times (in ms) for color-ring cues (upper panels) and color-word cues (lower panels) depending on stimulus-onset asynchrony (SOA), cue match, and validity. Error bars represent average standard errors

Errors were excluded, and RTs trimmed by the same criterion as in the prior experiments (in total 12.33%). A repeated-measures ANOVA, with the variables cue type (color-ring cue, color-word cue), validity (valid, invalid), cue match (matching, nonmatching), and SOA (150 ms, 700 ms), yielded a significant three-way interaction of Cue Type × Validity × Cue Match, *F*(1, 17) = 21.72, *p* < .001, ${\upeta}_{\mathrm{p}}^2$ = .56 (see Fig. 8). (For the remaining significant main effects and interactions, refer to Table 2 in Appendix B. For a complete listing of the mean correct RTs, see Table 7 in Appendix C.) Bonferroni-corrected post hoc *t* tests for eight different comparisons (alpha = .006) were conducted.

##### Color-word cues

We start with the tests of the color-word cue that now revealed a significant validity effect for the matching word cues, *t*(17) = −5.00, *p* < .001, *d* = −1.15, and an inverse validity effect for nonmatching color-word cues, *t*(17) = 4.85, *p* < .001, *d* = 1.11. This inverse validity effect for nonmatching word cues corresponds to a same-location cost and is a relatively common finding in contingent-capture experiments with feature-heterogeneous target displays, as were also used here (see Carmel & Lamy, 2015; Kerzel, 2019; Schoeberl et al., 2017).

##### Color-ring cues

In the post hoc tests, color-ring cues did not lead to any significant effects, all nonsignificant |*t*s|(17) < 3.12, all *p*s > .006, all |*d*s| < 0.72.

#### Accuracy analysis

**Fig. 9:**
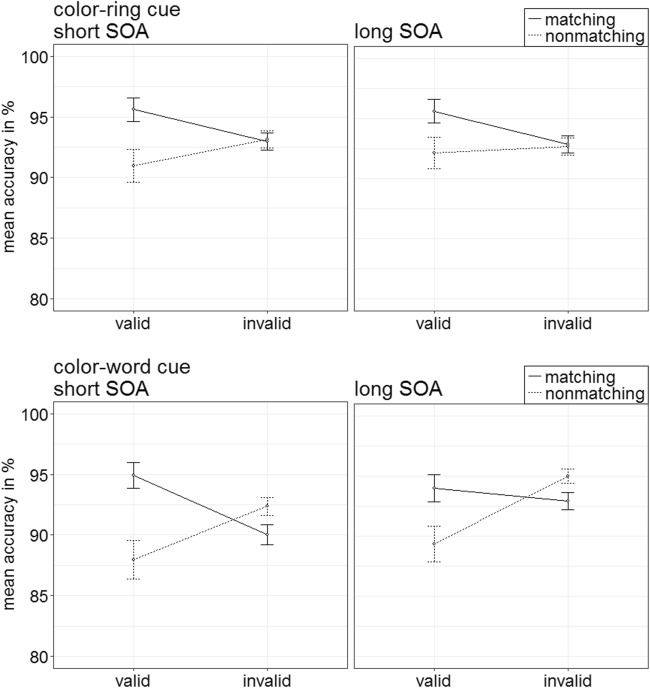
Experiment 2: Mean accuracy rates in percentages for color-ring cues (upper panels) and color-word cues (lower panels) depending on stimulus-onset asynchrony (SOA), cue match, and validity. Error bars represent average standard errors

Arcsine-transformed accuracy rates were submitted to a repeated-measures ANOVA similar to the one above. It showed a significant two-way interaction of Validity × Cue Match, *F*(1, 17) = 28.36, *p* < .001, ${\upeta}_{\mathrm{p}}^2$ = .63 (see Fig. 9). (See also Table 3 in Appendix B for further significant effects, and Table 7 in Appendix C for a complete list of the mean accuracy rate values.)

##### Color-word and color-ring cues

This time, data were not split up for cue type, as cue type did not significantly modulate the Validity × Cue Match interaction. With matching cues, Bonferroni-corrected post hoc *t* tests for four comparisons (alpha = .013) yielded significant validity effects, *t*(17) = 3.29, *p* = .004, *d* = 0.75, with better performance in valid (95.0%) than invalid (92.2%) trials. With nonmatching cues, performance was better in invalid (93.3%) than in valid (90.1%) conditions, *t*(17) = −3.97, *p* = .001, *d* = −0.91.

## Discussion

In Experiment 2, matching color-ring cues preceding color-word targets only elicited a small validity effect in the accuracies,^1^ but they did not elicit any validity effects in the RTs. The features of the color-ring cues were thus apparently too dissimilar to the templates by which participants searched for the color-word targets to reliably capture attention, thus confirming a major conclusion of Experiment 1: During top-down contingent capture, selection of color-ring cues mostly is not brought about by verbal/semantic templates.

In contrast, matching color-word cues preceding color-word targets elicited validity effects in RTs and accuracy rates, whereas nonmatching color-word cues prompted a same-location cost. Together, these effects prove that participants were able to read the parafoveally presented color-word cues or were at least able to identify some specifying features of the words, for example, specific shapes or letters. As matching cue and target words were also graphically similar to one another, the validity effects of the matching word cues might have also been due to a template for letters or for shapes. We picked up on the question of the exact reason for validity effects by matching color-word cues in Experiments 4 and 5.

For the time being, be this as it may. Importantly, the cueing effects of the matching words in Experiment 2 also demonstrated that the lack of any validity effect by the color-word cues in Experiment 1a was neither due to the inhibition of the irrelevant (nonmatching) color-word cues’ fonts, nor to the inconsistency between word meaning and font color, as both of these influences were present in Experiment 2, too.

We also found a same-location cost by the nonmatching color-word cues. Same-location costs are relatively common during search for color targets in heterogeneous target displays (cf. Eimer, Kiss, Press, & Sauter, 2009; Lamy, Leber, & Egeth, 2004), as were used here, although an agreement regarding their explanation could not be reached yet (cf. Carmel & Lamy, 2015). It is being discussed if same-location costs reflect an initial attentional capture and subsequent rapid disengagement of attention (Belopolsky, Schreij, & Theeuwes, 2010), a successful preemptive suppression of attentional capture (e.g., Eimer et al., 2009), more or less object-updating costs (e.g., Carmel & Lamy, 2014; see also Enns, Lleras, & Moore, 2009), or a mixture of (some of) these effects (Schoeberl et al., 2017). However, in the current study, which exact mechanism was responsible for the same-location costs in nonmatching color-word cue conditions was peripheral to the major research questions.

## Experiment 3

In Experiment 3, we put the hypothesis that verbal or semantic templates could be involved in contingent capture during color search to one more test. It is plausible that participants have used different top-down search sets in Experiments 1 and 2, as in each experiment only one type of stimulus was relevant for the search (either hue or color word) and as all cues were uninformative. Thus, the use of a search template for only hues in Experiments 1a and 1b or only words in Experiment 2 would have allowed for ignoring of the respective alternative cue type easily (i.e., word cues during search for hue targets and color-ring cues during search for word targets). In Experiment 3, to ensure that both stimulus types are relevant for the target search at the same time and thus encouraging the use of semantic templates to cover both target types, we presented both color and color-word targets that were pseudorandomly intermixed within the same blocks. If color-word cues lead to contingent capture effects regardless of the target type, we can conclude that participants’ search was based on semantic or verbal templates and the lack of such influences in Experiments 1 and 2 was due to the missing incentive for including higher level semantic processes in their search. Another possibility would be that both stimulus types—color-defined rings and verbally or semantically defined color words—are included in the top-down search templates. Studies have shown that two different features (e.g., two different colors) can be searched for at the same time in a top-down contingent manner (cf. Ansorge & Horstmann, 2007; Irons, Folk, & Remington, 2012). Research has also shown that if participants can search for two types of stimuli that would otherwise invite different templates (i.e., color-defined templates for red targets among white distractors; onset-directed templates for red onset targets) by only one template (in this case, for red targets), participants seemingly set up a single template allowing them to search for both of these target types at the same time. This is reflected in contingent-capture effects in such a situation—that is, validity effects of two types of different matching cues, red onset cues and red color cues, and successful ignorance of differently colored (blue) onset cues at the same time (see Experiment 5 of Goller et al., 2016). In the present experiment, our rationale was that an analogue principle during concomitant search for target words with a particular color meaning and hue-defined target rings of the same color as the meanings of the target words could amount to participants’ setup of a semantic template for the particular color meaning if that would allow them searching for both of the two different target types.

However, the question if contingent-capture effects can be based on one search template for two very different types of stimuli (here, for hues and color words) is generally underresearched (but see Büsel, Pomper, & Ansorge, 2018b; Irons et al., 2011), and the little existing evidence suggests that related kinds of top-down search for two features from different categories or dimensions (e.g., for a specific shape and a specific color) are not as efficient as search for two different features from the same category or dimension (i.e., for two different colors; Biderman, Biderman, Zivony, & Lamy, 2017). Experiment 3, thus, also aimed to investigate this topic, but as it is more peripheral to the major questions of the present study, the corresponding results are delegated to Appendix F.

Importantly, as target types were presented unpredictably intermixed, at the time that a color-word cue was presented, participants did not yet know the type of the following target. Therefore, the color-word cues could have always captured attention and—following this line—could have elicited contingent-capture effects. The interesting question is, if under these conditions, in which the meaning or verbal content (e.g., its phonology) of the matching color-word cues could be task-relevant and the processing of the matching color-word cues is ensured, we now find an interaction between contingent capture and target type that would be diagnostic of a different role of verbal or semantic templates during the processing of color-ring targets versus the processing of color-word targets.

### Method

#### Participants

Twenty-two psychology students (*M*_age_ = 21 years, *SD*_age_ = 4.02 years) of the University of Vienna participated in Experiment 3. Two students had to be excluded due to their error rates exceeding 20%.

#### Procedure and design

**Fig. 10:**
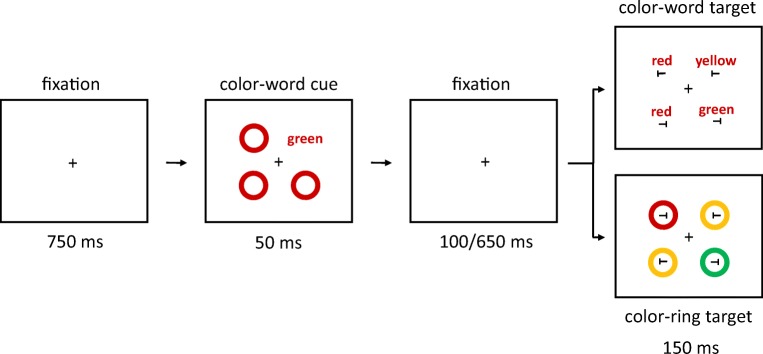
Example trial(s) of Experiment 3. The target is the color word *green* in the upper rightmost box (color-word target) or the green color ring in the lower rightmost box. The color-word cue in this example is matching and invalid. The arrows depict the flow of time. Stimuli are not drawn to scale. (Color figure online)

Participants searched for a specific color, which was equally likely implemented as a hue of a color-ring target (as in Experiments 1a and 1b) or as the meaning of a color-word target (as in Experiment 2), and where the hue of the color-ring targets and the meaning of the color-word targets referred to the same color. As in Experiment 1a, the color-ring targets were blue or green nonsingleton rings (with target color balanced across participants) presented among one red and two yellow distractor rings or among two red and one yellow distractor ring. As in Experiment 2, the color-word targets were the words *blue* and *green* (with word identity balanced across participants, but corresponding to the color of the searched-for color-ring targets) presented among the distractor words *yellow* and *red*, and all of these words were presented in red or in yellow fonts (see Fig. 10). Both types of targets, color-ring targets and color-word targets, were presented equally likely and intermixed within the same blocks. All of the targets were preceded by color-word cues, with the same cueing displays as were used in Experiment 1a (and Experiment 2).

### Results

#### Reaction-time analysis

**Fig. 11:**
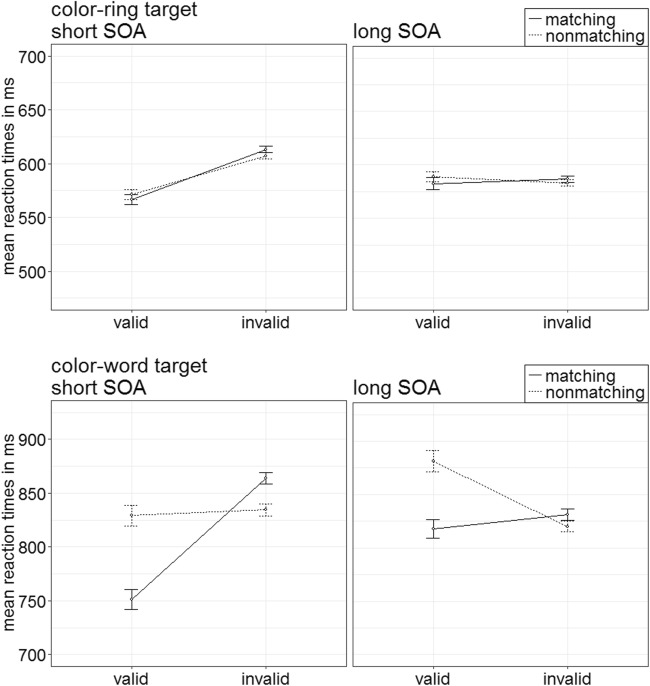
Experiment 3: Mean correct reaction times (in ms) for color-ring targets (upper panels) and color-word targets (lower panels) depending on stimulus-onset asynchrony (SOA), cue match, and validity. Error bars represent average standard errors. Note the different ranges of reaction times in the graphs for color-ring and color-word targets

Out of all trials, 13.64% were discarded from the RT analyses by the same criteria as were used in Experiments 1 and 2. This time, a repeated-measures ANOVA, with the variables target type (color-ring target, color-word target), validity (valid, invalid), cue match (matching, nonmatching), and SOA (150 ms, 700 ms), led to a significant three-way interaction of Target Type × Validity × Cue Match, *F*(1, 19) = 19.79, *p* < .001, ${\upeta}_{\mathrm{p}}^2$ = .51 (see Fig. 11). (Significant lower order interactions and main effects qualified by this significant highest order interaction of major interest can again be found in Table 2 in Appendix B; the exact mean correct RTs of all conditions are displayed in Table 8 in Appendix C.)

Post hoc *t* tests, Bonferroni corrected for eight comparisons (alpha = .006), examined validity effects and contingent capture for the different target types.

##### Color-ring targets

The *t* tests revealed a significant validity effect in color-ring target trials of both matching color-word cues (valid RT = 575 ms; invalid RT = 600 ms), *t*(19) = −6.16, *p* < .001, *d* = −1.34, and nonmatching color-word cues (valid RT = 580 ms; invalid RT = 595 ms), *t*(19) = −3.91, *p* = .001, *d* = −0.85.

##### Color-word targets

For word-target trials, we found the pattern corresponding to a classic contingent-capture effect: a validity effect of matching cues, *t*(19) = −5.70, *p* < .001, *d* = −1.24, and no validity effect of nonmatching cues, *t*(19) = 1.65, *p* = .115, *d* = 0.36.

An additional analysis of task-switch costs can be found in Appendix F.

#### Accuracy analysis

**Fig. 12:**
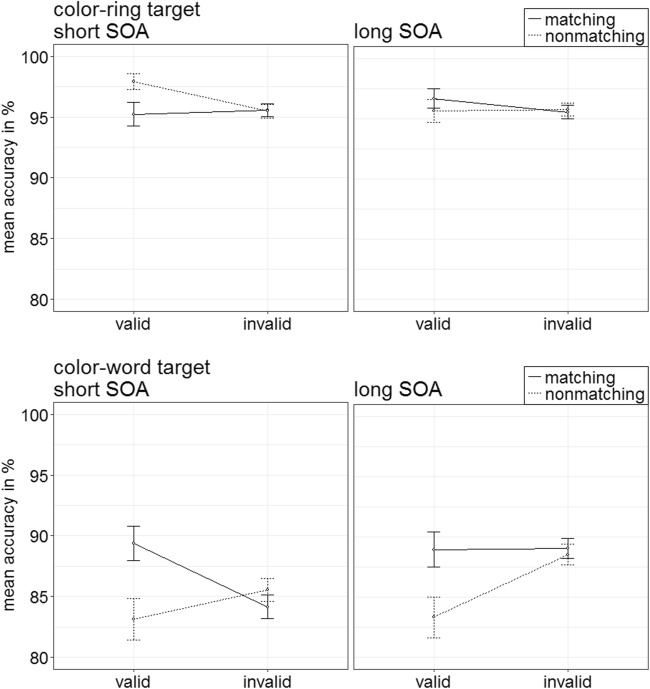
Experiment 3: Mean accuracy rates in percentages for color-ring targets (upper panels) and color-word targets (lower panels) depending on stimulus-onset asynchrony (SOA), cue match, and validity. Error bars represent average standard errors

Arcsine-transformed accuracy rates were analyzed by an ANOVA analogous to the first ANOVA of the RTs above. There was a significant three-way interaction of Target Type × Validity × Cue Match, *F*(1, 19) = 7.89, *p* = .011, ${\upeta}_{\mathrm{p}}^2$ = .29 (see Fig. 12). (See Table 3 in Appendix B for other significant main effects or interactions qualified by the three-way interaction, as well as Table 8 in Appendix C for an overview of the mean accuracy rates as a function of all factors.) Post hoc *t* tests, Bonferroni corrected for eight comparisons (alpha = .006), were split up for target types.

##### Color-word targets and color-ring targets

The post hoc tests yielded no significant validity effects for word-target trials, all |*t*s|(19) < 2.13, all *p*s = .046, all |*d*s| < 0.47, as well as no validity effects for color-ring target trials, all nonsignificant |*t*s|(19) < 1.45, all *p*s = .164, all |*d*s| < 0.32, but as is clear from Figs. 11 and 12, the results indicated the same pattern as in the RTs: numerically stronger validity-effect differences between matching and nonmatching color-word cues in color-word target trials than in color-ring target trials.

### Discussion

As in Experiment 2, color-word cues preceding color-word targets led to what we referred to as contingent-capture effects: Only top-down matching color-word cues seemingly captured attention and accordingly facilitated search in valid as compared with invalid conditions, whereas the nonmatching color-word cues elicited no validity effect. In contrast, in color-ring target trials, both matching and nonmatching color-word cues led to a significant validity effect. Importantly, as in Experiments 1 and 2, these results suggest again that searching for and processing specific color-ring targets is not based on verbal or semantic templates, but, this time, the lacking contingent-capture effect based on color-word cue meaning in the color-ring target trials was even observed under conditions that were definitely sensitive to color-word cues, as was reflected in their validity effects in color-ring target trials and their selective validity effect with matching color-word cues in color-word trials.

Apart from our main question—if search templates for colors are semantically or feature based—the fact that a validity effect of all color-word cues was found for the color-ring targets, regardless of the match (vs. nonmatch) of their meanings to the target colors, also sheds a light on the potential origin of the difference between the validity effect of the matching color-word cues and the lack of a validity effect of the nonmatching color-word cues in the color-word target trials. The interested reader should refer to Appendix F for a discussion of this topic.

Following Experiments 2 and 3, it is still open as to which mechanisms accounted for validity effects of matching color-word cues and the lacking validity effects of nonmatching color-word cues during word-target trials. It is possible that these differences between validity effects reflected word meaning. For example, if participants discriminated target words from distractor words by word meanings, it might have been more difficult to disengage attention from cues with a target-similar meaning. However, as each target word consisted of different letters or shapes for that matter and as even the same letter font was used for the matching cues as for the targets, it is possible that graphical shape representations accounted for the validity-effect difference. In addition, besides meaning, stronger validity effects of matching color-word cues than of nonmatching color-word cues might have also reflected their phonological representations. For example, more difficulties in disengaging from matching color-word cues might have been particularly likely if participants based their discrimination between target and distractor target-word representations held in working memory, as working memory representations are often of a phonological type (Baddeley, 1986; Baddeley, Lewis, & Valar, 1984). Experiments 4 and 5 therefore investigated these questions more thoroughly.

## Experiment 4

After Experiments 2 and 3, it is clear that matching color-word cues can lead to validity effects, but it is open which exact kind of mechanism was responsible for attention capture by matching cues and/or prolonged dwelling of attention at matching cues (see Appendix F for the latter possibility). So as not to suggest that only contingent capture based on search templates for specific words could account for the different validity effects of matching and nonmatching color-word cues, in the following, we used a more neutral terminology.^2^ For the rest of the present article, we speak of “target-distractor discrimination” to make clear that the processes responsible for validity effects of matching color-word cues and lacking or inverted cueing effects of nonmatching color-word cues might be preselective or postselective (defined as relative to the selection of any words).

**Fig. 13:**
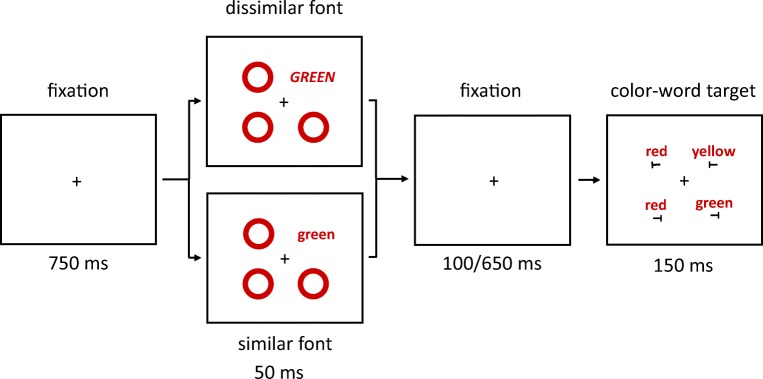
Exemplary trial(s) of Experiment 4. The target is the color word *green* in the rightmost box (the target word remained either *green* or *blue*, varied between participants, throughout the whole experiment). Two different cue versions are displayed in the boxes second from left (both invalid and matching): a color-word cue written in target-dissimilar font (top); a color-word cue written in target-similar font (bottom). The arrows depict the flow of time. Stimuli are not drawn to scale. (Color figure online)

Importantly, the discrimination between color-word targets and color-word distractors could be based on different characteristics, such as semantic, phonological, orthographical, or graphical information. Experiment 4 was the first of two experiments aimed at investigating the particular mechanisms that were involved in the discrimination between the color words and, by implication, in the robustness of the validity-effect differences between matching and nonmatching color-word cues. Due to the short presentation time of the color-word cues (50 ms) and the lack of evidence for semantic effects in Experiments 1–3, it is possible that participants did not read the color words but rather identified some specific graphical features to discriminate targets from distractors, like the shape of some of the letters of the targets. In Experiment 4, we tested if discrimination between color-word targets and color-word distractors could be based on graphical features by presenting color-word cues in target-similar or target-dissimilar fonts (see Fig. 13). If participants use the graphical features of the target words to tell target and distractors apart, only cues with the same graphical features as the target should capture attention and/or be difficult to ignore (and, thus, prevent rapid disengagement), and lead to validity effects. If graphical features are not used to tell targets from distractors, all matching word cues (e.g., with the same meaning and phonology as the target) could capture attention, independent of their graphical similarity to the target.

### Method

#### Participants

Twenty participants took part in Experiment 4 (*M*_age_ = 23.55 years, *SD*_age_ = 5.16 years).

#### Procedure and design

Methods were as in Experiment 2. In each trial, participants searched for a specific color word (*green* or *blue*, balanced across participants). However different from Experiment 2, the target display was equally likely and unpredictably preceded by either a color-word cue with a target-similar font (i.e., both target and cue were written in lowercase letters) or with a target-dissimilar font (i.e., the cue was in uppercase and the target in lowercase letters; see Fig. 13). (Color cue conditions were not realized.)

### Results

#### Reaction-time analysis

**Fig. 14:**
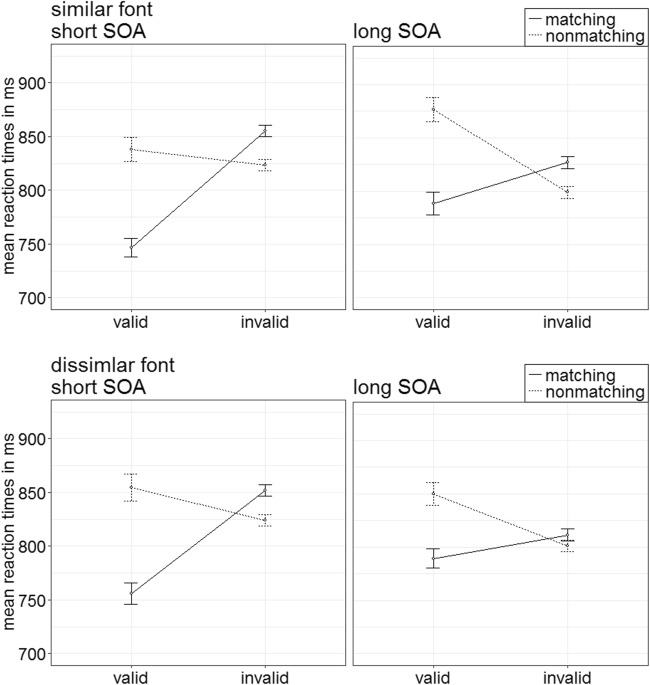
Experiment 4: Mean correct reaction times (in ms) for color-word cues with a font similar to the target (upper panels) and dissimilar to the target (lower panels) depending on stimulus-onset asynchrony (SOA), cue match, and validity. Error bars represent average standard errors

As in the first three experiments, trials with false responses were removed, as well as responses faster or slower than two standard deviations from the median per person and per condition (in total, 18.40%). A 2 × 2 × 2 × 2, repeated-measures ANOVA, with the factors cue type (color-word cue font similar to target, dissimilar to target), validity (valid, invalid), cue match (matching, nonmatching), and SOA (150 ms, 700 ms), was conducted. As its highest order significant interaction of major interest, it showed a two-way interaction of Validity × Cue Match, *F*(1, 19) = 88.10, *p* < .001, ${\upeta}_{\mathrm{p}}^2$ = .82 (see Fig. 14). (See also Table 2 in Appendix B for other significant main effects, and Table 9 in Appendix C for all mean RTs of Experiment 4.)

Post hoc *t* tests, Bonferroni corrected for four comparisons (alpha = .013), revealed a validity effect for matching cues (valid RT = 770 ms; invalid RT = 836 ms), *t*(19) = −5.26, *p* < .001, *d* = −1.15, and no significant effect for nonmatching cues (valid RT = 855 ms; invalid RT = 812 ms), *t*(19) = 1.30, *p* = .208, *d* = 0.28.

#### Accuracy analysis

**Fig. 15:**
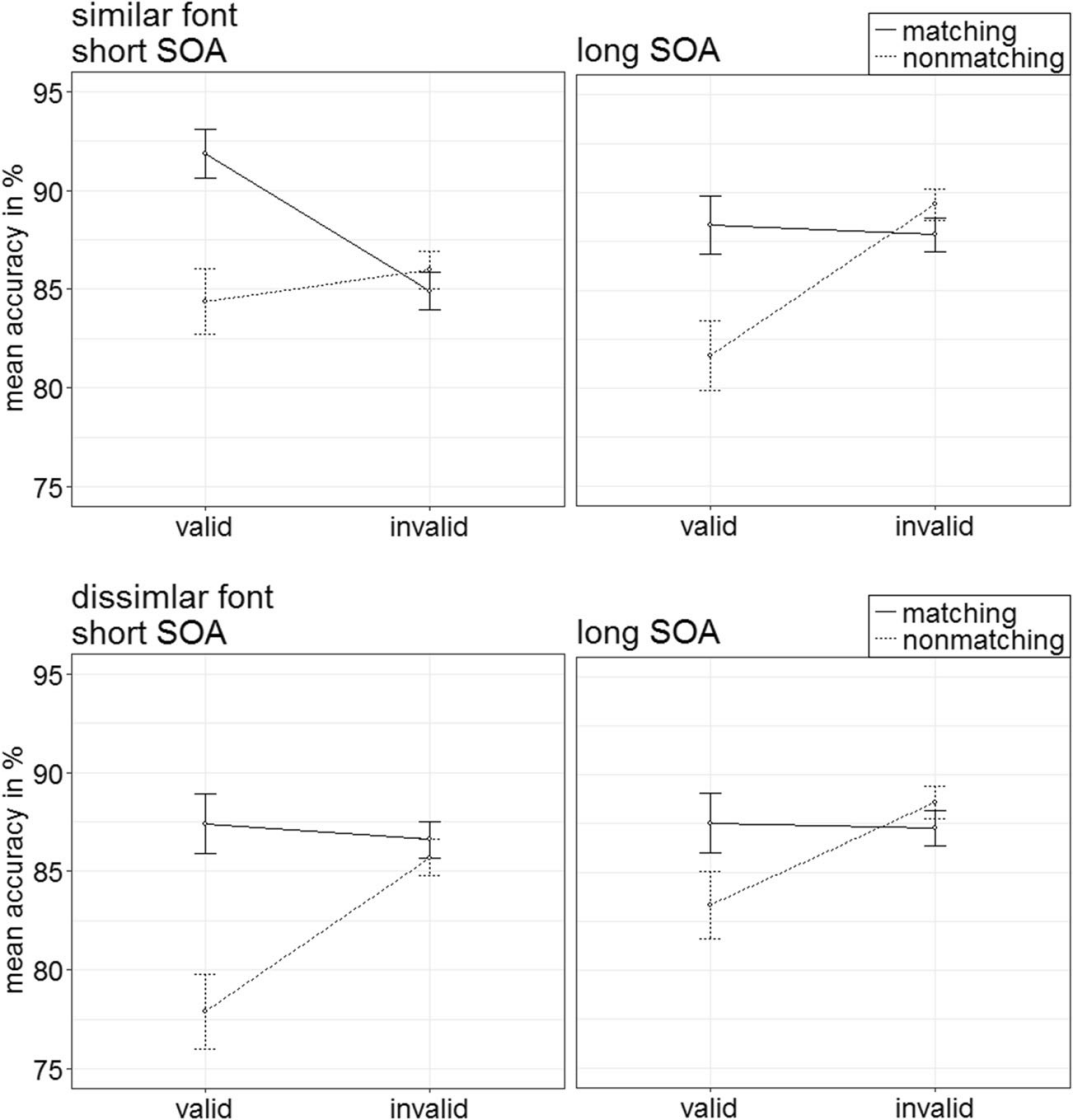
Experiment 4: Mean accuracy rates in percentages for color-word cues with a font similar to the target (upper panels) and dissimilar to the target (lower panels) depending on stimulus-onset asynchrony (SOA), cue match, and validity. Error bars represent average standard errors

As before, accuracy rates were arcsine transformed for an ANOVA as the one above. It also showed a significant two-way interaction of Validity × Cue Match, *F*(1, 19) = 55.64, *p* < .001, ${\upeta}_{\mathrm{p}}^2$ = .75 (see Fig. 15). (For other significant effects, refer to Table 3 in Appendix B; for all mean accuracy rates of Experiment 4, see Table 9 in Appendix C.)

Post hoc *t* tests with an alpha of .013 (Bonferroni corrected for four comparisons) yielded no significant validity effect for matching cues (valid accuracy rate = 88.8%; invalid accuracy rate = 86.7%), *t*(19) = 2.09, *p* = .050, *d* = 0.46. However, on average, reactions with nonmatching cues were significantly more accurate in invalid (87.4%) than in valid (81.8%) conditions, *t*(19) = −4.67, *p* < .001, *d* = −1.02.

### Discussion

In Experiment 4, validity effects were not influenced by the similarity between the font of the cue and that of the target. If the color-word cues were of the same meaning and phonology as the color-word targets, in valid conditions, both cue versions led to faster RTs than target-dissimilar cue words of a different meaning and phonology than the targets, and in invalid conditions, both more target-similar versions led to slower RTs than did more target-dissimilar cues. More target-dissimilar cues (with dissimilarity in terms of their meaning and phonology) of both font-similarity levels did not significantly influence RTs.

Slightly different effects arose in the accuracy rates: More target-similar cues did not lead to any significant effects, whereas for more target-dissimilar cues, performance was impaired in valid conditions compared with invalid conditions. This result corresponded to a same-location cost.

The difference of the validity effects suggested that color-word cues captured and/or held attention independently of their graphical similarity to the target words, falsifying the notion that target-distractor discrimination between color words was based on graphical features.

## Experiment 5

Experiment 5 was the second test of the mechanism of the discrimination between color-word targets and color-word distractors. Walenchok et al. (2016) found that phonological distractors sounding similar to searched for target objects delayed target search under some conditions, in which participants might have used phonologically defined search templates maintained in the phonological loop of working memory (cf. Baddeley, 1986). To examine the role of phonological representations as templates to discriminate between target-color and distractor-color words, in Experiment 5, participants again searched for a color-word target preceded by a color-word cue, but, additionally, in only one of two blocks we included an articulatory suppression task. This articulatory suppression task should interfere with rehearsal and processing via the phonological loop (Baddeley et al., 1984).

If discrimination between color words relies at least partly on phonological information held in working memory (Walenchok et al., 2016), validity effects of matching color-word cues should have been impaired when participants performed an additional articulatory suppression task. To equate attentional dual-task demands between blocks with articulatory suppression and control blocks without articulatory suppression, an additional secondary task that does not interfere with phonological processing was included in the control block: foot tapping (cf. Weywadt & Butler, 2013). Whatever the results in the phonological suppression task, in the control block, with the foot tapping as a secondary task, we expected to replicate validity effects of target-similar color-word cues and no validity effects of target-dissimilar color-word cues, as in Experiments 2 and 4, and in the color-word target conditions of Experiment 3.

### Method

#### Participants

Twenty-one students of the University of Vienna participated in Experiment 5 (*M*_age_ = 21.71 years, *SD*_age_ = 2.76 years). Three had to be excluded due to a high error rate (>20%).

#### Procedure and design

Methods were similar to those in Experiment 2. Participants searched for a predefined color-word target. However, a color-word cue preceded each target, and the experiment was divided into two blocks, with block order counterbalanced across participants. In the articulatory suppression block, participants searched for the target words, and their additional task was to repeat the syllable “fi” continuously to the beat of a metronome (60 bpm). In the control block, participants also had to search for color-word targets, but additionally they had to continuously tap with one foot, again to the beat of a metronome (60 bpm). This procedure was created following Saeki and Saito (2004). To ensure that the additional tasks were performed as instructed, audio files of speaking and tapping performance were recorded and checked for performance accuracy.

### Results

#### Reaction-time analysis

**Fig. 16:**
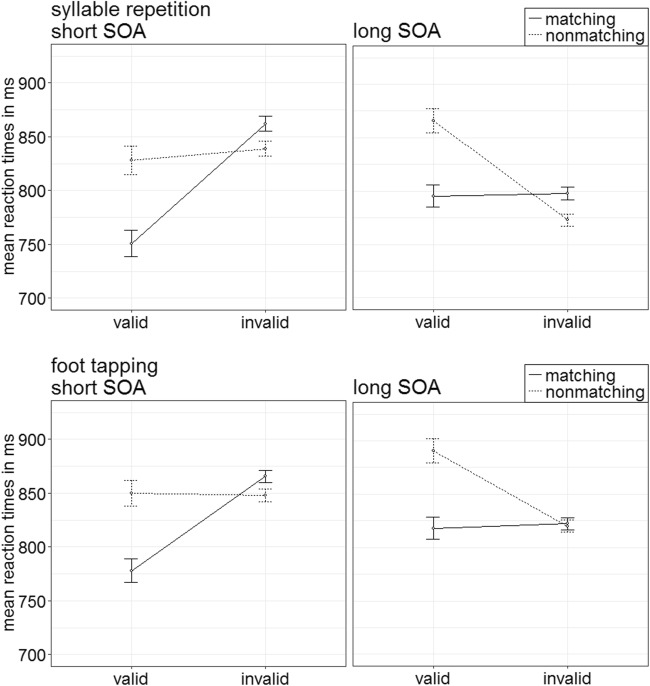
Experiment 5: Mean correct reaction times (in ms) for visual search plus syllable repetition (i.e., articulatory suppression task; upper panels) and visual search plus foot tapping (i.e., control task; lower panels) depending on stimulus-onset asynchrony (SOA), cue match, and validity. Error bars represent average standard errors

Trials were eliminated by the same criteria as in the preceding experiments (in total, 16.12%). A 2 × 2 × 2 × 2 repeated-measures ANOVA, with the variables block (visual search + foot tapping, visual search + syllable repetition), validity (valid, invalid), cue match (matching, nonmatching), and SOA (150 ms, 700 ms), demonstrated a significant two-way interaction of Validity × Cue Match, *F*(1, 17) = 68.84, *p* < .001, ${\upeta}_{\mathrm{p}}^2$ = .80 (see Fig. 16; Table 10 in Appendix C). (See Table 2 in Appendix B for further significant results.)

Post hoc *t* tests examining contingent-capture and validity effects with an alpha of .013 (Bonferroni corrected for four comparisons) revealed a significant validity effect for matching cues, *t*(17) = −6.48, *p* < .001, *d* = −1.49, and a significantly inverted validity effect for nonmatching cues, *t*(17) = 3.32, *p* = .004, *d* = 0.76.

#### Accuracy analysis

**Fig. 17:**
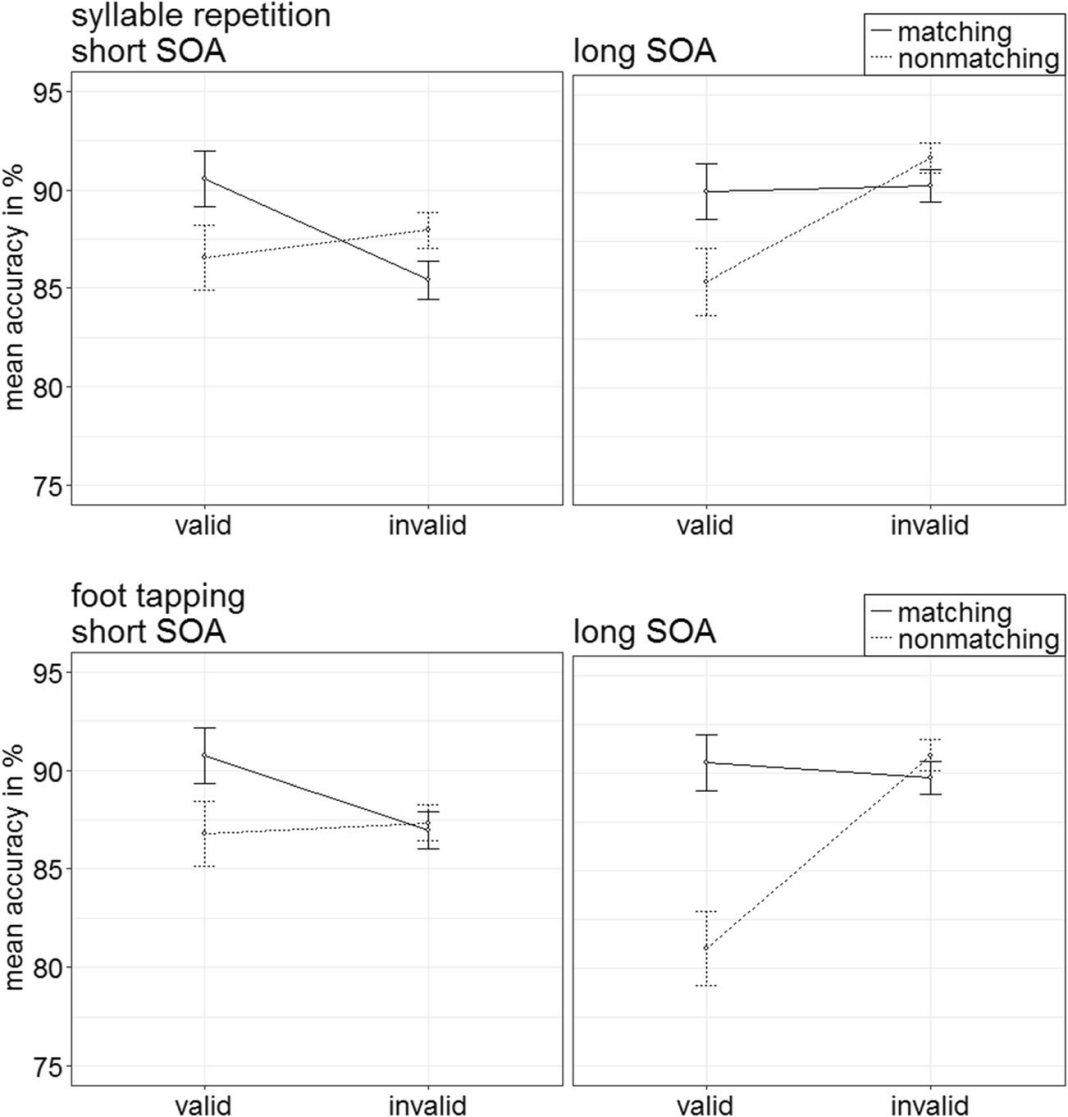
Experiment 5: Mean accuracy rates in percentages for visual search plus syllable repetition (i.e., articulatory suppression task; upper panels) and visual search plus foot tapping (i.e., control task; lower panels) depending on stimulus-onset asynchrony (SOA), cue match, and validity. Error bars represent average standard errors

Arcsine-transformed accuracy rates were submitted to an analogous ANOVA. It showed a significant two-way interaction of the variables Validity × Match, *F*(1, 17) = 14.10, *p* = .002, ${\upeta}_{\mathrm{p}}^2$ = .45 (see Fig. 17; Table 10 in Appendix C). (See Table 3 in Appendix B for further significant effects.)

Post hoc *t* tests, Bonferroni corrected for four comparisons (alpha = .013), yielded no significant validity effect for matching cues, *t*(17) = 2.34, *p* = .032, *d* = 0.54. However, on average, the reactions for nonmatching cues were significantly more accurate in invalid (89.5%) than in valid (85.0%) conditions, *t*(17) = −2.95, *p* = .009, *d* = −0.68.

### Discussion

Altogether, the results of Experiment 5 show validity effects by matching color-word cues, with weak same-location costs by nonmatching color-word cues. These effects were not affected by the articulatory suppression task at all. This is evidence against a target-distractor discrimination based on phonological features held in working memory. If discrimination would have relied at least partly on target-word rehearsal in the phonological loop, the articulatory suppression task should have interfered with the validity effect of the matching color-word cues. As this was not the case, it is more likely that representations for target-distractor discrimination were held in long-term memory or included other target-defining characteristics, such as orthographic features or, less likely, word meanings. To note, as the target was the same throughout the experiment, it is possible that participants used a long-term memory representation to discriminate between target and distractor words.

## General Discussion

### Contingent capture by color: No evidence for verbal and semantic templates

In a series of five experiments, we examined if top-down search could be based on verbal or semantic templates (for an overview, see Table 1). Different types of top-down search templates have been discussed in the visual search literature: templates consisting of visual features (e.g., Jenkins et al., 2017), verbal representations (e.g., Walenchok et al., 2016), or semantic characteristics (e.g., Belke et al., 2008). Using color-word cues, our results showed no evidence of contributions by verbal or semantic templates during search for color targets. In Experiment 1a, searching for a color-ring target, color-ring cues but not color-word cues led to contingent-capture effects. In fact, color-word cues had no significant validity effect at all. In Experiment 1b, color-word cues were replaced by parafoveally presented symbolic cues, each associated with a different specific color. As in Experiment 1a, only color-ring cues led to contingent-capture effects. Symbolic cues only produced an inversed validity effect in matching trials that could, however, be of a different origin (e.g., inhibition of the symbols because they otherwise elicited color representations at variance with 50% of the following targets). In Experiment 2, searching for color-word targets, matching but not nonmatching color-word cues led to validity effects, but now color-ring cues only created a small contingent-capture effect in the error rates.^1^ The lacking contingent-capture effect of the color-ring cues in the RTs showed again that capture by color-ring stimuli was mostly unrelated to word-directed (e.g., verbal or semantic) search templates. Finally, in Experiment 3, participants searched for both color-ring targets and color-word targets while only color-word cues were used. In this experiment, we studied the involvement of verbal or semantic templates during color search in the contingent-capture experiment, not by the contingent-capture effect of the color-ring or color-word cues themselves, but rather more indirectly by the interaction of cue type with the type of target. In this situation, the validity effect of the matching color-word cues was restricted to the color-word target trials. In contrast, in the color-ring target trials, both matching and nonmatching color-word cues showed a small validity effect. The latter was different from Experiment 1a. Again, however, the results were at odds with a verbal or semantic template during search for color targets, as this should have led to more capture by matching color-word cues than nonmatching color-word cues during search for color-ring targets, too.

Together, the results of Experiments 1a and 3 also suggested that the validity effect of color-word cues was absent where color words were irrelevant (in Experiment 1a) and that it was present where color words were relevant. This means that the validity effect of the color-word cues in the color-ring target trials of Experiment 3 reflected a form of contingent capture, too. However, this was a contingent-capture effect based on a search template for just any (color) word or (some of) its constituents (e.g., letters).

### Relation to the Stroop effect

Some of the results of Experiments 1a, 2, and 3 might appear surprising in light of Stroop effects and evidence for the automatic processing of color words during color discrimination tasks (MacLeod, 1991; MacLeod & McDonald, 2000; Stroop, 1935). For instance, responses are typically faster to print colors of color words with a color-congruent meaning than with a color-incongruent meaning, and such Stroop effects can also be observed where there is an interval between color word and color stimulus (e.g., Dyer & Severance, 1973; Weekes & Zaidel, 1996) and where color word and color stimulus are not presented at the same location (e.g., Dyer, 1973; Gatti & Egeth, 1978; Kahneman & Chaiczyk, 1983). Hence, some of the critical side conditions for a Stroop effect were fulfilled in the present experiments, and this raises the question as to why no influence of word identities or of the interaction between word identities and spatial word-to-color-ring target distance on color-ring target processing was found in these experiments.

One decisive difference between the present study and past research is that, typically, the Stroop effect is measured where stimulus color needs to be discriminated by different overt responses. Response interference between incompatible color-word meaning and target colors therefore contributes to the standard Stroop effect (Durgin, 2000; Klein, 1964). However, no overt responses directly discriminated between target colors in the current study. Instead, the current study used a compound search task in which color was necessary to locate the target, but the overt responses discriminated a different attribute of the same targets—here, the orientation of a *T* at color-ring target location. In addition, the Stroop effect diminishes for task-peripheral and to-be-suppressed distractors (Wuehr & Frings, 2008), and, arguably, our participants suppressed all of the uninformative cues of the present study, only with different success (i.e., less success with top-down matching cues) (Fuchs et al., 2013; Gaspelin, Ruthruff, & Lien, 2016). These factors might have prevented any strong Stroop effects in the present study.

### Mechanisms involved in the discrimination of color words

In the final two experiments, we also studied how target and distractor words were discriminated so that more evidence for capture and/or dwelling of attention could be found with matching than with nonmatching color-word cues. Several possibilities of how participants could have discriminated between the parafoveally presented visual color words are conceivable, such as a discrimination between graphical features of the (target vs. distractor) color words, between phonological features of these words, or even between their semantic features. As some of these variants would not even have required reading of the words but could have rather invited shape-based search, we tested some of these possibilities. For example, past studies have shown that parafoveally presented stimuli that are graphically similar to the target word facilitate target word processing (Rayner, McConkie, & Zola, 1980; Slattery, Angele, & Rayner, 2011; see also Paap, Newsome, & Noel, 1984). This implies that discrimination based on the graphical features of the parafoveally presented target words and, therefore, the matching and nonmatching color-word cues was a possibility, so we put this to a test in Experiment 4. Here, we tested if target-color words could be discriminated based on graphical features, but whether a target-similar or target-dissimilar font was used for the color-word cues had no impact on the validity effect of the matching color-word cues. However, this finding does not preclude the possibility that the participants could have used some sort of orthographical template, as even parafoveally presented stimuli that were not identical with the target and only graphically similar to the target have been shown to facilitate target word processing (Rayner et al., 1980; Slattery et al., 2011; see also Paap et al., 1984).

In addition, phonological target-word representations could have likewise allowed discriminating between target and distractor words and, hence, also between matching and nonmatching color-word cues (cf. Baddeley, 1986; Walenchok et al., 2016). In Experiment 5, we tested if color-word discrimination could be based on subvocal rehearsal, but whether we used a secondary articulatory suppression task or a secondary tapping control task had no influence on the validity effect of the matching color-word cues. This does preclude that a phonological working memory representation was responsible for the validity effect of the matching color-word cues. However, such discrimination based on phonological target-word characteristics could be based on long-term memory (cf. Coltheart, Curtis, Atkins, & Haller, 1993). In line with the possibility that participants phonologically discriminated between target and distractor words of the current Experiments 2–5, some prior studies demonstrated phonological priming by parafoveally presented words in a preview advantage of target-word recognition (e.g., Pollatsek, Lesch, Morris, & Rayner, 1992). In addition, as the target words were kept the same for each participant throughout Experiments 2–5, favorable conditions for templates based on long-term memory could have been met in the current study (Carlisle et al., 2011; see also Grubert, Carlisle, & Eimer, 2016). Thus, phonological long-term memory templates could have possibly been responsible for stronger validity effects following matching than nonmatching color-word cues during search for color-word targets.

In theory, it is even possible that participants used a semantic representation to discriminate between target and distractor words. In line with this possibility, in German, as was used here, semantic facilitation by parafoveally presented words is a robust effect (Hohenstein & Kliegl, 2014; Hohenstein, Laubrock, & Kliegl, 2010). Now, seemingly, Experiments 2 and 3 did not support a semantic discrimination. Otherwise, we should have found more evidence of a contingent-capture effect of color-ring cues during search for color-word targets and of color-word cues during search for color-ring targets because interactions between sensory stimuli and words are typical, if not even defining, characteristics for a semantic effect (cf. Glaser & Glaser, 1989; McCauley, Parmelee, Sperber, & Carr, 1980; Schmidt & Zelinsky, 2009). However, it is possible that more evidence of a semantic discrimination during search for color-word (and even color-ring) targets could be found if stimuli less complex than words or outline sketches of objects semantically associated to colors (e.g., of hearts for red or stars for yellow stimuli; see Experiment 1b) would be used as cues. For example, one could try to use spoken words as auditory cues in future experiments (cf. Lupyan & Spivey, 2010). Maybe semantics of spoken words are easier to process and, thus, able to tap into the semantic nature of color-directed search templates.

### Relation to categorical search

In the current study, the lack of contingent-capture effects by color-word cues during search for color-ring targets could have been due to some form of category-search template constraining the overall range of stimuli that could capture attention (cf. Wyble, Folk, & Potter, 2013; Yang & Zelinsky, 2009). For example, searching for a color-ring target, it might be difficult to successfully ignore other stimuli partly matching the target category, such as other rings. Yet it could be that stimuli of a sufficiently different category, such as words or shapes that are not rings, would never capture attention in this situation. If this was the case, our results would be suggestive that category-based capture effects (i.e., capture by objects similar to the target category rather than to specific target features) are not brought about by a category’s semantics. If the latter were true, again, we would have expected capture by color-word cues during search for color-ring targets, as cross-modal processing (here, across visual and verbal modes) is one hallmark of a semantic effect (e.g., Glaser & Glaser, 1989). However, what we said above applies here, too, and future tests could use simpler stimuli than visual words or than outline sketches semantically associated to particular colors, to once more put to test the possibility that search for color targets is based on target semantics.

## Conclusion

Altogether, our results are best in line with hue-based contingent-capture effects. As participants treated color-ring cues and color-ring targets, on the one hand, and color-word cues and color-word targets, on the other hand, as largely separate events (see also Appendix F, switch costs of Experiment 3) and as only color-ring cues elicited clear contingent-capture effects when the targets were out of the same stimulus category (namely, also colored rings), verbal or semantic templates during search for color in contingent-capture experiments were not supported by the present findings. In addition, which types of discrimination between relevant and irrelevant words accounted for stronger validity effects by matching than nonmatching word cues in color-word target trials requires additional research in the future, but semantic and phonological working memory templates are unlikely candidates for color-word based contingent-capture effects.

This, however, does not mean that we would have generally falsified the use of semantic templates in visual search. Factors, such as how well an irrelevant cue can be filtered out by a hue-based template, could have fostered participants’ use of a hue-based rather than a semantic template in some conditions (e.g., the present contingent-capture Experiments 1a and 1b). Thus, we can only conclude that contingent capture by uninformative color cues is likely not based on semantic search templates.

## Open practices statement

None of the data or stimuli material of Experiments 1a–5 are available online; however, we will gladly provide them if requested. The experiments were not preregistered.

### Author note

This research was supported by WWTF grant (CS15-001) awarded to Soonja Choi and Ulrich Ansorge (University of Vienna).

## Appendix A

Detailed description of the differences in the results between a two standard deviation and three standard deviation cutoff value in the preprocessing of the RTs

Together with the removal of trials with wrong responses, a three standard deviation cutoff value leads to a total removal of 6.63% (Experiment 1a), 9.17% (Experiment 1b), 8.13% (Experiment 1b, congruency test), 9.66% (Experiment 2), 9.79% (Experiment 3), 14.62% (Experiment 4), and 12.57% (Experiment 5) of the data. The pattern of results remains the same, except for the following differences.

Experiment 1a: In the post hoc *t* tests after the significant four-way interaction in the ANOVA, the validity effect of matching word cues in the short SOA becomes significant, whereas the validity effect of matching color cues in the long SOA is no longer significant.

Experiment 1b: An additional two-way interaction in the ANOVA is significant, SOA × Validity. In the post hoc *t* tests justified by the significant three-way interaction, the validity effect of matching symbolic cues in the short SOA is no longer significant.

Experiment 3: In the ANOVA, the main effect of validity as well as the Target Type × SOA interaction are no longer significant. In the post hoc *t* tests examining the significant three-way interaction, the difference between matching and nonmatching invalid color targets becomes significant, whereas the same-location costs with nonmatching color targets, as well as the difference between matching and nonmatching invalid color-word target conditions, are no longer significant. In the additional analysis examining task-switch costs, the three-way interaction Target Type × Validity × Target Type in Trial *N* − 1 is significant. The post hoc *t* test examining the difference between target type and target type in trial *n* − 1 showed significant task-switch costs in all conditions, except for invalid target words.

Experiment 4: In the post hoc *t* tests, the same-location costs of nonmatching cues are now significant.

## Appendix B

Detailed listing of all significant results of ANOVAs of mean correct reaction times and accuracy rates of Experiments 1a–5

**Table 2:** All significant main effects and interactions of the conducted analyses of variance with mean correct reaction times of Experiments 1a–5

Significant main effects and interactions	*df*	*F*	*p*	${\upeta}_p^2$
Experiment 1a				
Cue Type	20	7.56	.012	.27
Validity	20	15.67	<.001	.44
Cue Type × SOA	20	10.24	.004	.34
Cue Type × Validity	20	139.60	<.001	.87
Validity × Cue Match	20	39.71	<.001	.67
Cue Type × SOA × Validity	20	13.40	.002	.40
Cue Type × SOA × Cue Match	20	14.17	.001	.41
Cue Type × Validity × Cue Match	20	53.15	<.001	.73
Cue Type × SOA × Validity × Cue Match	20	17.75	<.001	.47
Experiment 1b				
Validity	19	12.99	.002	.41
Cue Type × Validity	19	78.27	<.001	.80
Validity × Cue Match	19	65.23	<.001	.77
Cue Type × SOA × Validity	19	13.47	.002	.41
Cue Type × SOA × Cue Match	19	7.28	.014	.28
Cue Type × Validity × Cue Match	19	47.01	.001	.71
SOA × Validity × Cue Match	19	9.00	.007	.32
Cue Type × SOA × Validity × Cue Match	19	10.15	.005	.35
Experiment 2				
Cue Type	17	5.41	.033	.24
Cue Match	17	36.84	<.001	.68
SOA × Validity	17	37.25	<.001	.69
Cue Type × Cue Match	17	14.45	.001	.46
Validity × Cue Match	17	52.23	<.001	.75
Cue Type × SOA × Validity	17	25.47	<.001	.60
Cue Type × Validity × Cue Match	17	21.2	<.001	.56
Experiment 3				
Target Type	19	514.06	<.001	.96
Validity	19	6.82	.017	.26
Cue Match	19	22.50	<.001	.54
Target Type × SOA	19	4.96	.038	.21
SOA × Validity	19	39.35	<.001	.67
Target Type × Cue Match	19	15.83	<.001	.45
Validity × Cue Match	19	38.16	<.001	.67
Target Type × SOA × Validity	19	8.39	.009	.31
Target Type × Validity × Cue Match	19	19.79	<.001	.51
Experiment 4				
Cue Match	19	47.12	<.001	.71
Cue Type × SOA	19	4.98	.038	.21
SOA × Validity	19	13.15	.002	.41
Validity × Cue Match	19	88.10	<.001	.82
Experiment 5				
Cue Match	17	31.15	<.001	.65
SOA × Validity	17	100.62	<.001	.86
Validity × Cue Match	17	68.84	<.001	.80

**Table 3:** All significant main effects and interactions of analyses of variance with mean accuracy rates of Experiments 1a–5

Significant main effects and interactions	*df*	*F*	*p*	${\upeta}_p^2$
Experiment 1a				
Validity	20	20.27	<.001	.50
Cue Type × Validity	20	29.04	<.001	.59
Cue Type × Validity × Cue Match	20	23.18	<.001	.54
SOA × Validity × Cue Match	20	12.38	.002	.38
Experiment 1b				
Validity	19	28.01	<.001	.60
Cue Type × Validity	19	11.62	.003	.38
Cue Type × SOA × Validity	19	29.60	<.001	.61
Cue Type × Validity × Cue Match	19	5.89	.025	.24
Cue Type × SOA × Validity × Cue Match	19	5.41	.031	.22
Experiment 2				
Validity	17	5.74	.028	.25
Cue Match	17	11.45	.004	.40
Validity × Cue Match	17	28.36	<.001	.63
Experiment 3				
Target Type	19	118.45	<.001	.86
Target Type × Cue Match	19	7.19	.015	.27
Target Type × Validity × Cue Match	19	7.89	.011	.29
Experiment 4				
Cue Match	19	24.93	<.001	.57
SOA × Validity	19	7.22	.015	.28
Validity × Cue Match	19	55.64	<.011	.75
Experiment 5				
Cue Match	17	8.40	.001	.33
SOA × Validity	17	15.26	.001	.47
Validity × Cue Match	17	14.10	.002	.45

**Table 4:** All significant main effects and interactions of the conducted analysis of variance with mean correct reaction times of Experiments 3 including the additional fifth factor target type in trial *n* – 1

Significant main effects and interactions	*df*	*F*	*p*	${\upeta}_p^2$
Target Type	19	524.14	<.001	.97
Validity	19	7.19	.015	.27
Cue Match	19	22.37	<.001	.54
Target Type × SOA	19	4.52	.047	.19
SOA × Validity	19	40.38	<.001	.68
Target Type × Cue Match	19	15.01	.001	.44
Validity × Cue Match	19	37.97	<.001	.67
Target Type × Target Type in Trial *N*-1	19	53.44	<.001	.74
Target Type × SOA × Validity	19	7.74	.012	.29
Target Type × Validity × Cue Match	19	19.51	<.001	.51

## Appendix C

Listing of mean correct reaction times and accuracy rates of Experiments 1a–5 as a function of the respective factors

**Table 5:** Reaction times (RTs; in ms) and accuracy rates (ACCs; in %) of Experiment 1a as a function of the factors cue type, stimulus-onset asynchrony (SOA), cue match, and validity

Experiment 1a					
Cue type	SOA	Cue match	Validity	RT	ACC
Color-ring cue	Short SOA	Matching	Valid	499	97.8
			Invalid	586	92.6
		Nonmatching	Valid	555	97.6
			Invalid	554	94.8
	Long SOA	Matching	Valid	546	98.0
			Invalid	582	93.2
		Nonmatching	Valid	562	95.2
			Invalid	563	95.7
Color-word cue	Short SOA	Matching	Valid	575	90.5
			Invalid	552	96.3
		Nonmatching	Valid	561	96.2
			Invalid	554	95.0
	Long SOA	Matching	Valid	558	95.4
			Invalid	558	96.0
		Nonmatching	Valid	567	94.8
			Invalid	559	96.2

**Table 6:** Reaction times (RTs; in ms) and accuracy rates (ACCs; in %) of Experiment 1b as a function of the factors cue type, stimulus-onset asynchrony (SOA), cue match, and validity

Experiment 1b					
Cue type	SOA	Cue match	Validity	RT	ACC
Color-ring cue	Short SOA	Matching	Valid	525	96.9
			Invalid	643	88.7
		Nonmatching	Valid	592	94.8
			Invalid	591	91.3
	Long SOA	Matching	Valid	579	93.5
			Invalid	629	91.8
		Nonmatching	Valid	603	92.9
			Invalid	597	93.0
Symbolic cue	Short SOA	Matching	Valid	608	92.3
			Invalid	580	95.1
		Nonmatching	Valid	590	92.9
			Invalid	574	93.5
	Long SOA	Matching	Valid	599	92.7
			Invalid	598	90.8
		Nonmatching	Valid	609	92.7
			Invalid	602	93.3

**Table 7:** Reaction times (RTs; in ms) and accuracy rates (ACCs; in %) of Experiment 2 as a function of the factors cue type, stimulus-onset asynchrony (SOA), cue match, and validity

Experiment 2					
Cue type	SOA	Cue match	Validity	RT	ACC
Color-ring cue	Short SOA	Matching	Valid	802	95.6
			Invalid	822	93.0
		Nonmatching	Valid	832	91.0
			Invalid	811	93.1
	Long SOA	Matching	Valid	817	95.6
			Invalid	827	92.8
		Nonmatching	Valid	844	92.1
			Invalid	817	92.7
Color-word cue	Short SOA	Matching	Valid	740	94.9
			Invalid	875	90.0
		Nonmatching	Valid	855	88.0
			Invalid	834	92.3
	Long SOA	Matching	Valid	814	94.0
			Invalid	821	92.9
		Nonmatching	Valid	920	89.4
			Invalid	793	95.0

**Table 8:** Reaction times (RTs; in ms) and accuracy rates (ACCs; in %) of Experiment 3 as a function of the factors target type, stimulus-onset asynchrony (SOA), cue match, and validity

Experiment 3					
Target Type	SOA	Cue Match	Validity	RT	ACC
color-ring target	short SOA	matching	valid	567	95.2
			invalid	613	95.6
		nonmatching	valid	571	97.9
			invalid	607	95.5
	long SOA	matching	valid	582	96.7
			invalid	587	95.5
		nonmatching	valid	589	95.6
			invalid	583	95.8
color-word target	short SOA	matching	valid	751	89.4
			invalid	864	84.2
		nonmatching	valid	829	83.1
			invalid	834	85.6
	long SOA	matching	valid	818	89.0
			invalid	831	89.0
		nonmatching	valid	881	83.3
			invalid	820	88.5

**Table 9:** Reaction times (RTs; in ms) and accuracy rates (ACCs; in %) of Experiment 4 as a function of the factors cue type, stimulus-onset asynchrony (SOA), cue match, and validity

Experiment 4					
Cue type	SOA	Cue match	Validity	RT	ACC
Dissimilar font	Short SOA	Matching	Valid	756	87.4
			Invalid	852	86.6
		Nonmatching	Valid	855	77.9
			Invalid	824	85.7
	Long SOA	Matching	Valid	789	87.5
			Invalid	811	87.3
		Nonmatching	Valid	850	83.3
			Invalid	801	88.5
Similar font	Short SOA	Matching	Valid	747	91.9
			Invalid	855	84.9
		Nonmatching	Valid	838	84.4
			Invalid	823	86.0
	Long SOA	Matching	Valid	788	88.3
			Invalid	827	87.8
		Nonmatching	Valid	877	81.7
			Invalid	799	89.4

**Table 10:** Reaction times (RTs; in ms) and accuracy rates (ACCs; in %) of Experiment 5 as a function of the factors block, stimulus-onset asynchrony (SOA), cue match, and validity

Experiment 5					
Block	SOA	Cue match	Validity	RT	ACC
Syllable repetition	Short SOA	Matching	Valid	751	90.6
			Invalid	862	85.4
		Nonmatching	Valid	828	86.6
			Invalid	839	88.0
	Long SOA	Matching	Valid	795	90.0
			Invalid	798	90.3
		Nonmatching	Valid	866	85.4
			Invalid	773	91.7
Foot tapping	Short SOA	Matching	Valid	778	90.7
			Invalid	866	87.0
		Nonmatching	Valid	850	86.8
			Invalid	848	87.3
	Long SOA	Matching	Valid	818	90.5
			Invalid	822	89.7
		Nonmatching	Valid	891	81.0
			Invalid	820	90.9

## Appendix D

Listing of post hoc comparisons of RTs between valid matching and nonmatching cues, as well as between invalid matching and nonmatching cues, from Experiments 1a–5

### Experiment 1a

Reflecting the contingent-capture effects, a selective validity effect for matching and its absence for nonmatching color-ring cues was found in the short SOA: RTs were faster in valid matching than valid in nonmatching (555 ms) conditions, *t*(20) = −9.53, *p* < .001, *d* = −2.03, and RTs were slower in invalid matching than in invalid nonmatching (554 ms) conditions, *t*(20) = 6.28, *p* < .001, *d* = 1.34. With the long SOA, in invalid conditions, RTs for nonmatching cues (563 ms) were also significantly faster than for matching cues, *t*(20) = 3.81, *p* = .001, *d* = 0.81. There was no significant difference between valid matching and nonmatching color-ring cues, *t*(20) = −2.32, *p* = .031, *d* = −0.49. Color-word cues did not produce any significant effects, all |*t*s|(20) < 2.26, all *p*s > .035, all |*d*s| < 0.48.

### Experiment 1b

For color-ring cues, in the short SOA condition, RTs were faster in valid matching than in nonmatching (592 ms) conditions, *t*(19) = −6.99, *p* < .001, *d* = −1.53, and RTs were slower in invalid matching than in nonmatching (591 ms) conditions, *t*(19) = 6.51, *p* < .001, *d* = 1.42. The same pattern arose in the long SOA, faster RTs after valid matching than after nonmatching cues (603 ms), *t*(19) = −4.00, *p* < .001, *d* = −0.87, as well as slower RTs after invalid matching than after nonmatching cues (597 ms), *t*(19) = 5.57, *p* < .001, *d* = 1.22. For symbolic cues, no further effects were found, all nonsignificant, |*t*s|(19) < 2.12, all *p*s > .048, all |*d*s| < 0.46.

### Experiment 2

Reflecting contingent-capture effect, for color-word cues, RTs were also faster in valid matching (776 ms) than valid nonmatching (888 ms) conditions, *t*(17) = −7.57, *p* < .001, *d* = −1.74, and slower in invalid matching (847 ms) than in invalid nonmatching (813 ms) conditions, *t*(17) = 6.58, *p* < .001, *d* = 1.51. For color-ring cues, no further effects were significant, all |*t*s|(17) < 2.38, all *p*s > .029, all |*d*s| < 0.55.

### Experiment 3

Related to the same nonsignificant influence of cue match on validity, in color-ring target trials, there was neither a significant difference between valid matching and valid nonmatching conditions, nor between invalid matching and invalid nonmatching conditions, all nonsignificant |*t*s|(19) < 2.77, all *p*s > .012, all |*d*s| < 0.60. In color-word target trials, the validity effect difference was reflected in faster RTs for valid matching (785 ms) than for valid nonmatching (855 ms) conditions, *t*(19) = −5.12, *p* < .001, *d* = −1.12, and slower RTs for invalid matching (847 ms) than for invalid nonmatching (827 ms) conditions, *t*(19) = 3.97, *p* = .001, *d* = 0.87.

### Experiment 4

In valid conditions, we found faster RTs for matching than for nonmatching cues, *t*(19) = −5.93, *p* < .001, *d* = −1.29, and in invalid conditions slower RTs for matching than for nonmatching cues, *t*(19) = 3.74, *p* = .001, *d* = 0.82.

### Experiment 5

In valid conditions, RTs were faster for matching (786 ms) than for nonmatching (858 ms) cues, *t*(17) = −7.50, *p* < .001, *d* = −1.72. In invalid conditions, RTs were slower for matching (836 ms) than for nonmatching (819 ms) cues, *t*(17) = 3.92, *p* =.001, *d* = 0.90.

## Appendix E

Post hoc comparisons of ACCs between valid matching and nonmatching cues, as well as between invalid matching and nonmatching cues, from Experiments 1a–5

### Experiment 1a

With color-ring cues—reflecting the contingent-capture effect—in invalid conditions, more errors were made with matching cues than with nonmatching cues (accuracy rate: 95.2%), *t*(20) = −3.60, *p* = .002, *d* = −0.77. Only, the better performance for matching than for nonmatching cues in valid conditions was not significant, *t*(20) = 1.97, *p* = .063, *d* = 0.42. For color-word cues, there were no significant effects, all |*t*s|(20) < 2.13, all *p*s > .046, all |*d*s| < 0.45.

### Experiment 1b

No further significant effects were found, neither for color-ring cues, all |*t*s|(19) < 2.08, all *p*s > .051, all |*d*s| < 0.45, nor for symbolic cues, all |*t*s|(19) < 2.79, all *p*s > .012, all |*d*s| < 0.61.

### Experiment 2

Related to the contingent-capture effect, in the valid condition, fewer errors were made with a matching cue than with a nonmatching cue, *t*(17) = 4.96, *p* < .001, *d* = 1.14. In the invalid condition, there was no significant difference in performance between matching and nonmatching cues, *t*(17) = −2.74, *p* = .014, *d* = −0.63.

### Experiment 3

There were no further significant effects, either with color-word targets, all |*t*s| (19) < 3.02, all *p*s > .007, all |*d*s| < 0.66, or with color-ring targets, all |*t*s|(19) < 0.44, all *p*s > .664, all |*d*s| < 0.10.

### Experiment 4

For valid conditions, there was a significantly higher accuracy after matching than after nonmatching cues, *t*(19) = 7.46, *p* < .001, *d* = 1.63, but in invalid conditions, there was no significant difference in performance for matching versus nonmatching cues, *t*(19) = −1.05, *p* = .307, *d* = −0.23.

### Experiment 5

In valid conditions, we found significantly higher accuracies with matching (90.5%) than with nonmatching cues, *t*(17) = 4.15, *p* = .001, *d* = 0.95. However, in invalid conditions, there was no significant difference for matching (88.1%) versus nonmatching cues, *t*(17) = −1.57, *p* = .135, *d* = −0.36.

## Appendix F

Additional analysis and discussion of Experiment 3

### Additional analysis of task-switch costs in Experiment 3

To investigate if the trial-by-trial change of target type required a change of the search template, we looked for switch costs between alternative task representations (cf. Monsell, 2003)—here, between different search templates. We also tested if this kind of template switching could have affected the degree to which the cues captured attention. Maybe attention capture by the matching color-word cues was stronger following a target-word trial than following a color-ring target trial. Therefore, an ANOVA of the correct mean reaction times with target type (color-word target vs. color-ring target), validity (valid vs. invalid), cue match (matching vs. nonmatching), and with an additional fifth variable target type in trial *n* – 1 (word target in trial *n* – 1 vs. color-ring target in trial *n* – 1) was conducted. The results remained unchanged, except for a further significant two-way interaction of target type in *n* and target type in *n* – 1, *F*(1, 19) = 53.44, *p* < .001, ${\upeta}_{\mathrm{p}}^2$ = .74, reflecting switch costs (see Table 4 in Appendix B for a complete listing of all significant main effects and interactions). Where the target type changed across subsequent trials, participants reacted significantly slower than where it repeated, both for color-ring targets, *t*(19) = 5.92, *p* < .001, *d* = 1.29 (change: 603 ms, repeat: 583 ms), and color-word targets, *t*(19) = 3.90, *p* < .001, *d* = 0.85 (change: 839 ms, repeat: 826 ms). The alpha level was Bonferroni corrected for two comparisons (α = .025).

### Discussion of the nonselective validity effect of color-word cues in Experiment 3

Overall faster responses in color-ring target conditions than in color-word target conditions (see Fig. 11; Table 8 in Appendix C) suggested that, at an early stage of processing, all color-word cues captured attention, regardless of the match of their meaning to the specific target templates, and that a stronger validity effect of the matching than of the nonmatching color-word cues in color-word target trials must have subsequently built up across time from this initial general word-based validity effect. According to this reasoning, only in the case of the slower responses to the color-word targets would sufficient time have passed so that the slower top-down contingency based on color-word meaning did show up (see also X. Wu, Liu, & Fu, 2016).

This pattern of results would be in line with Theeuwes, Atchley, and Kramer’s (2000) rapid disengagement model. According to this explanation, any salient singleton (here, the color-word cue) initially captures attention, and subsequently only the less target-similar nonmatching cue, but not the more target-similar matching cue, would be suppressed sufficiently quickly, so that ultimately only the more difficult to suppress capture by the matching cues would be reflected in a validity effect (see also Gaspelin et al., 2016).^3^ However, different from Theeuwes et al.’s rapid disengagement model, in the current study, early capture by all color-word cues was most likely not due to the bottom-up salience of the color-word cues but rather reflected a form of top-down contingency. In the present study, validity effects by the color-word cues were not observed during search for color-ring targets in Experiment 1a, in which participants did not also search for color-word targets at the same time and in which the color-word cues were therefore task irrelevant. Yet a true bottom-up capture effect by the salient color-word cues should have shown up in Experiment 1a, too, regardless of the task relevance of the color words. Thus, it is likely that the validity effect of all color-word cues in the color-ring target trials of the present experiment reflected a type of top-down contingent capture—for example, a general sensitivity of top-down templates during search for only particular color words for either letters, words, color meanings, or a combination of these.

This conclusion was also supported by an additional analysis of the RTs in which we tested if trial-by-trial switches from one target type to the other target type led to a switch cost indicative of separate search templates for the two targets. Although this analysis indicated that search was faster where the same types of targets were used in subsequent trials than where the type of target had switched from the preceding trial, the validity effect of the word cues was not further affected by this (cf. Büsel, Pomper, et al., 2018a; Irons et al., 2011). This ruled out that word-cue capture was due to intertrial priming by word targets in *n* – 1 (cf. Theeuwes, 2013, 2018). If priming by word targets in trial *n* – 1 would have been responsible for word-cue capture, we would have expected the word cues’ validity effects to be stronger or selectively present following a word-target trial. As the interaction between target type in *n* – 1 and validity was far from significant, *F*(1, 19) = 72.37, *p* = .140, we can rule out an intertrial priming account, in turn supporting a top-down interpretation of the word cues’ validity effect.

The remaining switch costs following a trial-by-trial change of the target types thus also suggested that the search sets responsible for the cue-word validity effect were not the same as those that participants ultimately used to select the word targets. Maybe participants first searched for letters or for words, regardless of their meanings, accounting for the validity effect of all word cues, and only then participants also scrutinized word meanings, allocating attention more at meaning-matching cues but rejecting cues that did not match the target words’ meaning. On top of that, the onset of a color-ring target display might have led to the efficient suppression of all words.

To note, however, conditions in Experiment 3 were special in that half of all trials allowed relatively swift color-ring search instead of slower color-word search. Maybe a two-step, top-down strategy of first selecting all words in a top-down way, and only initiating a discrimination between target and distractor words when this was necessary, was encouraged by the fact that very often a deeper going processing of the words was not required (i.e., in the color-ring target trials of Experiment 3). Therefore, we do not want to rule out that contingent capture by matching color-word cues only, and based on search templates for only particular color-word targets, is at work where each and every trial requires searching for a particular word—that is, under conditions as in Experiment 2.

## Footnotes

1 This comparison is an additional analysis, as the significant main effect of validity is a lower order effect and cannot be interpreted as part of the omnibus ANOVA. The corresponding validity effect of color-ring cues on accuracies during search for color-word targets might be due to the activation of sensory representations (here, of colors) during reading or processing of color-word target meanings (as required per task).

2 To note, however, a number of results confirmed that the contingent-capture account holds true for color cues. For example, when using the N2pc—an event-related potential record of attention capture with millisecond precision that allows to study capture directly after the cues before the overt responses—one can see that the matching cue captures attention initially, directly after its onset, more reliably than the nonmatching cue, thus ruling out a deallocation explanation, which assumes initially equal capture by all cues but faster disengagement of attention following nonmatching than matching cues (Ansorge et al., 2011; Eimer & Kiss, 2008).
